# The interplay between physical cues and mechanosensitive ion channels in cancer metastasis

**DOI:** 10.3389/fcell.2022.954099

**Published:** 2022-09-07

**Authors:** Kaustav Bera, Alexander Kiepas, Yuqi Zhang, Sean X. Sun, Konstantinos Konstantopoulos

**Affiliations:** ^1^ Department of Chemical and Biomolecular Engineering, The Johns Hopkins University, Baltimore, MD, United States; ^2^ Johns Hopkins Institute for NanoBioTechnology, The Johns Hopkins University, Baltimore, MD, United States; ^3^ Department of Biomedical Engineering, The Johns Hopkins University, Baltimore, MD, United States; ^4^ Department of Mechanical Engineering, The Johns Hopkins University, Baltimore, MD, United States; ^5^ Department of Oncology, The Johns Hopkins University, Baltimore, MD, United States

**Keywords:** mechanosensitive (MS) ion channel, cancer metastasis, physical forces, cell migration, cell cytoskeleton

## Abstract

Physical cues have emerged as critical influencers of cell function during physiological processes, like development and organogenesis, and throughout pathological abnormalities, including cancer progression and fibrosis. While ion channels have been implicated in maintaining cellular homeostasis, their cell surface localization often places them among the first few molecules to sense external cues. Mechanosensitive ion channels (MICs) are especially important transducers of physical stimuli into biochemical signals. In this review, we describe how physical cues in the tumor microenvironment are sensed by MICs and contribute to cancer metastasis. First, we highlight mechanical perturbations, by both solid and fluid surroundings typically found in the tumor microenvironment and during critical stages of cancer cell dissemination from the primary tumor. Next, we describe how Piezo1/2 and transient receptor potential (TRP) channels respond to these physical cues to regulate cancer cell behavior during different stages of metastasis. We conclude by proposing alternative mechanisms of MIC activation that work in tandem with cytoskeletal components and other ion channels to bestow cells with the capacity to sense, respond and navigate through the surrounding microenvironment. Collectively, this review provides a perspective for devising treatment strategies against cancer by targeting MICs that sense aberrant physical characteristics during metastasis, the most lethal aspect of cancer.

## Introduction

Cells possess complex mechanisms to tightly regulate the molecular machinery involved in proliferation, migration and apoptosis. In contrast, cancer cells frequently lack critical checkpoints and/or homeostatic mechanisms, resulting in sustained proliferation and/or decreased apoptosis, which lead to the formation of primary tumors and enhanced invasion into the surrounding stroma, thereby often culminating in the formation of metastases (Hanahan and Weinberg, 2011). Traditionally, genetic modifications and biochemical factors have been implicated in transforming resident cells into metastatic cancer cells. More recently, however, physical cues have emerged as critical mediators of this transformation (Emon et al., 2018).

The extracellular matrix (ECM) provides biochemical and biophysical cues which can influence cancer progression and metastasis. The ECM is an intricate three-dimensional (3D) network composed of proteoglycans and fibrous proteins, such as collagens, elastins, fibronectins and laminins, which provides structural support to tissues (Winkler et al., 2020). These fibers provide critical attachment points for cells to facilitate the proper development and organization of tissues (Rozario and DeSimone, 2010). Additionally, the ECM provides docking sites for cytokines, growth factors, and other bioactive molecules which promote the growth, differentiation and maintenance of cells by engaging intracellular signaling pathways (Taipale and Keski-Oja, 1997; Schonherr and Hausser, 2000). Aberrant changes in ECM structure and organization can perturb tissue homeostasis by promoting abnormal cell proliferation, elevated contractility, and transformation due to transcriptional changes (Wozniak et al., 2003; Paszek et al., 2005; Ondeck et al., 2019; Stowers et al., 2019). As tumor cells proliferate, the surrounding ECM undergoes further architectural changes which impact cellular physiology, resulting in a sustained feedforward loop that contributes to disease progression (Bonnans et al., 2014; Winkler et al., 2020).

**FIGURE 1:**
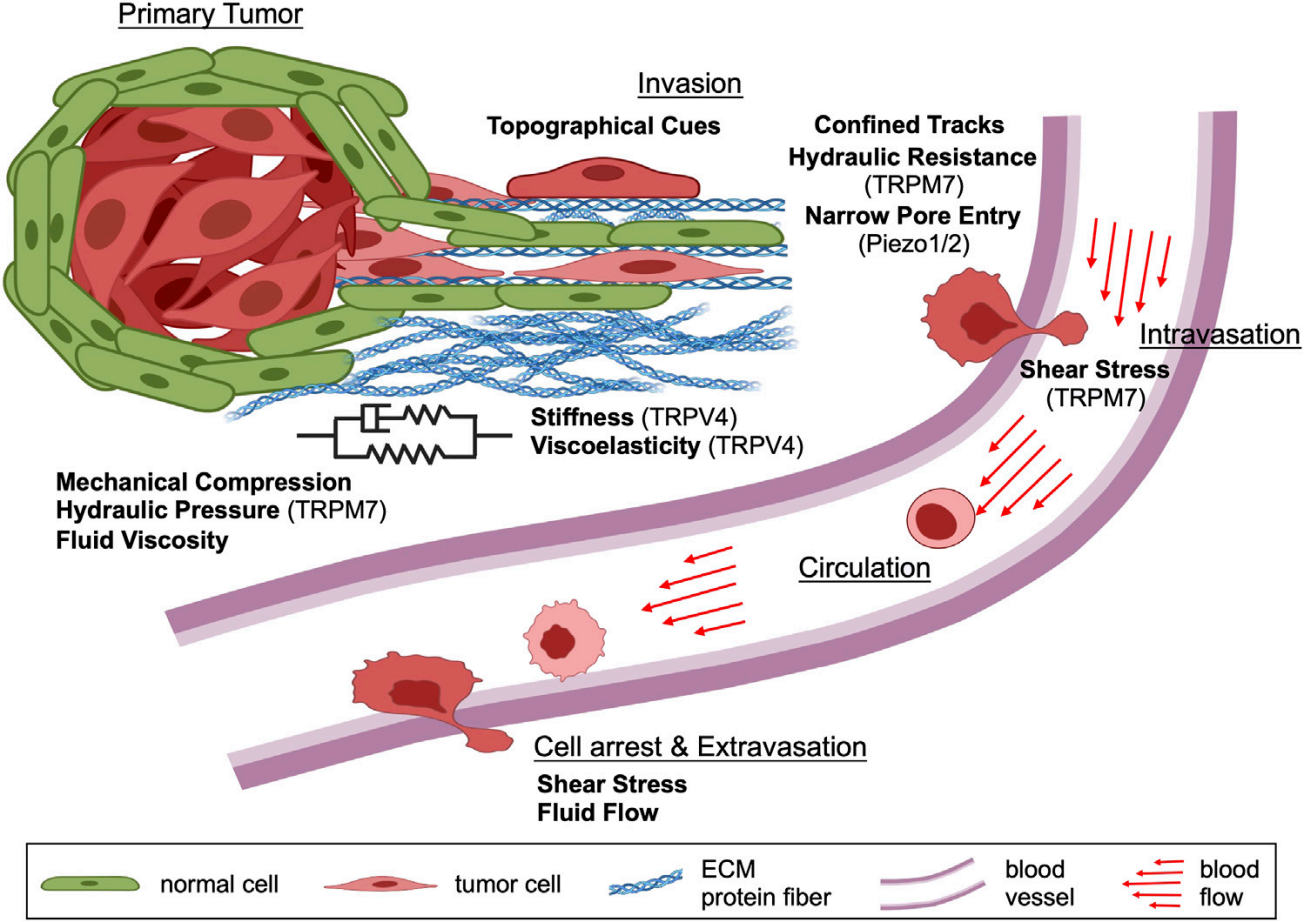
Schematic depicting the contribution of various physical cues during different steps of the metastatic cascade. In the primary tumor, cancer cells experience mechanical compression and hydraulic pressure as well as different levels of substrate stiffness, viscoelasticity and extracellular fluid viscosity. Following primary tumor cell dissemination, invading cancer cells encounter substrate stiffness and viscoelasticity, mechanical compression, fluid viscosity, solid barriers, confined tracks, and other topographies within the local tissue microenvironment. During intravasation, cancer cells experience shear stress caused by blood flow and continue to be exposed to this physical cue while in circulation. The viscosity of blood is also elevated relative to interstitial fluids, which potentially impacts cell behavior. As cancer cells arrest to the vascular endothelium and extravasate out of the bloodstream, they experience shear stress from the blood flow. Taken together, cancer cells are exposed to many different physical forces during the metastatic cascade which they must sense, integrate and interpret to engage appropriate cellular mechanisms for efficient dissemination. Schematic created with BioRender.com.

Cancer cells in the primary tumor experience mechanical compression, hydraulic pressure, fluid viscosity, substrate stiffness and viscoelasticity (Wisdom et al., 2018; Nia et al., 2020; Park et al., 2020) (Table 1). As cells disseminate from the primary tumor and invade into the local tissue microenvironment, they encounter additional mechanical forces due to the organization and structure of the ECM. Cancer cells must navigate a variety of topographical features, such as ECM fibers, small pores and channel-like tracks with prescribed physical properties to reach the vasculature (Paul et al., 2017). Fiber architecture and cross-linking density regulate ECM porosity, stiffness and viscoelasticity (Yamada and Sixt, 2019; Chaudhuri et al., 2020). Extracellular fluid, which hydrates the ECM, also exerts osmotic pressure and hydraulic resistance (Table 1) due to hydrostatic pressure and fluid viscosity on migrating cells (Li et al., 2020a). Once cancer cells enter the vasculature, they must withstand shear stress caused by blood flow to disseminate throughout the body (Shen and Kang, 2020). Thus, cancer cells must sense, integrate and interpret a multitude of physical forces during each step of the metastatic cascade to successfully colonize distant organs/tissues (Figure 1).

**TABLE 1:** Physical cues encountered by cancer cells during the metastatic cascade.

Physical force	Description
Compression	Pushing force applied to a structure. A growing tumor can apply pushing forces on the surrounding tissue, which in turn also causes compression on the tumor
Contact guidance	Describes the tendency of cells to change their orientation based on surrounding geometrical patterns, such as nano/microgrooves on substrates or the direction of ECM fibers
Elasticity	Describes the ability of a material to resist deformation and return to its original size and shape when external forces are removed. A material that is fully elastic recovers its size and shape immediately after the applied load is removed
Hydraulic resistance	Fluid pressure felt by a moving object from the surrounding fluid, which must flow to accommodate the object movement. Many factors can influence hydraulic resistance, including the viscosity of the surrounding fluid, the geometry in which the fluid is enclosed, and the presence of any obstacles to flow
Interstitial Fluid Pressure (IFP)	A pushing force exerted on an object immersed in interstitial fluid due to the presence of hydraulic pressure. The hydraulic pressure can arise from osmotic pressure in the fluid and also hydrostatic pressure from the presence of gravity. Osmotic pressure is the force arising from the entropy of mixing water with other solutes, which drives water flow from a low to a high concentration compartment, thereby increasing hydraulic pressure in the high concentration compartment
Shear stress	Shear stress arises from friction between layers of moving fluids or between solid and fluid layer interfaces. Shear stress can be experienced by a body floating in a fluid stream or along the wall of a fluid conduit, such as a blood vessel wall
Stiffness	The ratio between the applied force and the deformation of the object experiencing force. A stiffer object requires more applied force to achieve the same deformation
Tension	Pulling or stretching force exerted upon a structure. Tension is the opposite of compression. Stretching force on the membrane (tension) around an ion channel can activate the ion channel
Topography	Describes the arrangement of physical features that affect the roughness of a surface, including curvature, columns, grooves, and other nano/micro factors
Turbulent Flow	Fluid motion characterized by random fluctuations in pressure and velocity due to the irregular movement of fluid particles. In contrast, laminar flow describes the smooth movement of fluid in parallel layers with no disruptions
Viscoelasticity	Describes both the elastic and viscous properties of a material. Most biological materials are viscoelastic, rather than elastic, and exhibit a time-dependent delay in deformation and relaxation in response to external forces. Viscoelastic materials also dissipate a fraction of energy it took to deform them, resulting in some permanent deformation after external forces are removed
Viscosity	Describes the resistance of a fluid to change shape at a given flow rate (i.e., resistance to flow) due to internal friction between molecules in the fluid

Mechanosensitive ion channels (MICs), or stretch-activated ion channels, are a family of pore-forming proteins that play an important role in sensing and transducing physical stimuli. Their cell surface localization often places them among the first few molecules to sense external physical cues and initiate intracellular biochemical cascades in a process known as mechanotransduction (Patel et al., 2010; Ranade et al., 2015; Canales Coutiño and Mayor, 2021). To sense complex physical cues and changes within the microenvironment, MICs facilitate Ca^2+^ entry into cells through a mechanism known as channel gating (Table 2). This process requires a threshold level of membrane tension to initiate a conformational change from a closed to open state. The gating process can also depend on transmembrane voltage, which is regulated by ion channels (Yellin et al., 2018; Li et al., 2020a). Mechanosensitive gating mechanisms can be broadly categorized into 2 types: 1) direct mechanosensing, which is regulated by physical changes in the cell membrane, and 2) indirect mechanosensing, which is regulated by intracellular signaling cascades (Patel et al., 2010; Ranade et al., 2015; Petho et al., 2019).

**TABLE 2:** Mechanosensitive ion channels and their gating mechanisms.

Ion channel	Structure and mechanism of activation
Piezo 1/2	High-resolution cryo-electron microscopy (EM) studies reveal that Piezo1/2 forms a homotrimeric complex with a cationic permeable pore (Ge et al., 2015). Specifically, mouse Piezo1 has a three-bladed, propeller-shaped structure with three long intracellular beams that pivot together like a lever in response to mechanical force (Saotome et al., 2018; Zhao et al., 2018). Further studies combining cryo-EM with high-speed atomic force microscopy demonstrate that Piezo1 undergoes a reversible, flattening deformation when force is applied (Lin et al., 2019), effectively opening the central pore for cation-selective permeation (Coste et al., 2012; Lin et al., 2019). Recent studies employing biochemical assays also indicates that Piezo1 channel can be mechanically gated by the actin cytoskeleton *via* a cadherin-β-catenin-vinculin complex, thus suggesting that a force-from-filament model can work together with force-from-lipid model (Wang et al., 2022). However, further characterization needs to be performed, especially on the molecular-structural front, to determine how exactly such forces are transmitted. Piezo channels also possess an inactivation gate that is sensitive to membrane voltage and prevents further mechanical stimulation until channels are reset by outward permeation (Moroni et al., 2018)
TRPC1	TRPC1 is activated through intracellular signaling pathways that involve G-protein-coupled receptors, phospholipase C (PLC) and IP_3_ or the depletion of intracellular Ca^2+^ stores (Clapham, 2003; Trebak et al., 2007; Baudel et al., 2020). Namely, STIM1 senses Ca^2+^ depletion in the endoplasmic reticulum and recruits Orai1 channels in the plasma membrane to replenish Ca^2+^ stores (Cheng et al., 2011). Local increases in Ca^2+^ concentration due to Orai1-mediated Ca^2+^ influx subsequently stimulate TRPC1 trafficking and insertion into the plasma membrane (Cheng et al., 2011). Once localized to the plasma membrane, TRPC1 directly contributes to store-operated Ca^2+^ entry by facilitating Ca^2+^ influx in response to STIM1 gating (Yuan et al., 2007; Cheng et al., 2011)
TRPC5	TRPC5 channels are activated by elevated levels of extracellular Ca^2+^, La^3+^ and Gd^3+^ and G-protein coupled receptors (Clapham, 2003; Blair et al., 2009; Ningoo et al., 2021). In the latter pathway, PLC-mediated hydrolysis of phosphatidylinositol 4,5-bisphosphate (PIP_2_) into diacylglycerol also activates protein kinase C (PKC), which directly controls the sensitivity of TRPC5 to PIP_2_ *via* phosphorylation (Ningoo et al., 2021). TRPC5 does not exhibit a strong preference for Ca^2+^ and also passes Na^+^ (Clapham, 2003)
TRPM2	TRPM2 assembles into a tetramer with a three-tiered architecture: the top tier contains S1-S6 transmembrane and TRP helices, the middle tier contains an MHR4 domain and the rib helix, while the bottom tier contains an N-terminal MHR1/2 domain and a C-terminal NUDT9H domain that is gated by ADP ribose, a metabolic product of NAD (Wang et al., 2018b; Huang et al., 2018). Upon ADP ribose binding, NUDT9H undergoes a conformational change that triggers the rotation of MHR1/2. Ca^2+^ binding subsequently tilts the TRP helix, twists the MHR and rotates the gating S6 helix to open the channel (Wang et al., 2018b; Huang et al., 2018). TRPM2 conducts Ca^2+^ and Na^+^ with equal permeability (Clapham, 2003)
TRPM4	TRPM4 forms a homotetrameric channel with multiple transmembrane and cytosolic domains, which assemble into a three-tiered architecture: the top tier contains S1-S6 transmembrane and the TRP domain like TRPM2, the middle tier contains a linker helical domain with 12 helices (LH1-LH12), while the bottom tier contains an N-terminal nucleotide-binding domain, an ankyrin repeat domain and a C-terminal coiled-coil helix (Guo et al., 2017; Duan et al., 2018a). The N-terminal nucleotide-binding domain and the C-terminal coiled-coil regulate tetrameric assembly of the channel (Guo et al., 2017). ATP binding to TRPM4 causes a conformational change in the nucleotide-binding domain that disrupts the bottom-tier tetrameric assembly and inhibits channel activity (Guo et al., 2017; Duan et al., 2018a). Calmodulin and PKC phosphorylation can also modulate the sensitivity of TRPM4 (Nilius et al., 2005). On the other hand, channel gating may be controlled by the C-terminal coiled coil domain, which can slide within the central hole like a piston (Guo et al., 2017). Unlike other TRP channels, TRPM4 is impermeable to divalent cations. Instead, TRPM4 has a selectivity filter for monovalent cations that preferentially permits Na^+^ in response to PIP_2_ and Ca^2+^ (Duan et al., 2018a)
TRPM7	The quaternary structure of TRPM7 is similar to other TRPM channels with a few notable differences (Duan et al., 2018b). TRPM7 possesses a short helix between S2 and S3 that is one helical turn shorter than in TRPM2 and TRPM4 (Duan et al., 2018b). This change may affect channel gating upon changes in membrane bilayer composition and thickness. The selectivity filter of TRPM7 also differs from TRPM4 by one amino acid, which is essential for divalent cation permeation of Ca^2+^ and Mg^2+^ (Duan et al., 2018b). Furthermore, the hydrophobic loops and two helices belonging to the N-terminal domain are anchored to the inner leaflet of the plasma membrane in TRPM7 (Duan et al., 2018b). The movement of this region may produce additional conformational changes, resulting in large vertical and rotational motions of the N-terminal domain. Finally, the C-terminus of TRPM7 contains a protein kinase domain, which is proteolytically cleaved in a cell-type-specific manner, translocates to the nucleus, and phosphorylates histones leading to changes in gene expression (Krapivinsky et al., 2014). In addition to Ca^2+^ and Mg^2+^, TRPM7 is permeable to other divalent cations and monovalent cations (Krapivinsky et al., 2014). TRPM7 is constitutively active, however its activity is strongly repressed by intracellular concentrations of Mg^2+^, Ba^2+^, Sr^2+^, Zn^2+^ Mn^2+^, Cl^−^ and Br^−^, low intracellular pH, Mg-nucleotides, polyamines, and PIP_2_ hydrolysis (Fleig and Chubanov, 2014)
TRPV2	TRPV2 is a TRPV channel subfamily member that forms a tetrameric Ca^2+^-permeable cation channel. The central ion pathway is formed by transmembrane segments S5-S6, which are flanked by S1-S4 voltage-sensor-like domains (Liao et al., 2013; Huynh et al., 2016) from adjacent subunits. Like other TRP channels, TRPV2 contains two constrictions or gates: an upper gate resides at the selectivity filter in the outer pore region while a lower gate is comprised of the distal end of S6 (Huynh et al., 2016). Both upper and lower gates are closed in the apo state, whereas the presence of agonists causes gate opening (Huynh et al., 2016). Unlike true voltage-gated ion channels, the S1-S4 domain remains static during channel activation; however, it provides an external surface for ligand binding (Liao et al., 2013; Huynh et al., 2016). Channel opening occurs following a series of conformational changes that begin with an upward shift of the intracellular ankyrin repeat domain. The pore helix subsequently exhibits a clockwise twist which causes allosteric changes in the upper and lower gate (Huynh et al., 2016; Zubcevic et al., 2016). Detailed structural analysis suggests that TRPV2 can accommodate partially hydrated Ca^2+^, Na^+^ and K^+^ ions, as well as large organic cations, in the apo state (Huynh et al., 2016). Conversely, PIP_2_ and ERK phosphorylation regulate the functions of TRPV2 (Santoni et al., 2020)
TRPV4	TRPV4 forms a symmetric tetramer like TRPV2 with six transmembrane domains (S1-S6); however, S4-S5 linker adopts an ordered loop structure than an ɑ-helix (Deng et al., 2018). In typical voltage-gated ion channels, the ɑ-helix functions are a mechanical lever to couple voltage-sensor activation and pore opening. Consequently, the absence of this linker in TRPV4 results in a different gating mechanism. The S1-S4 domain rotates ∼90° counter-clockwise around the S4 helix, which moves S3 towards the central pore and creates an extensive packing interface with S5-S6 of the adjacent subunit (Deng et al., 2018). In this unique conformation, S3 intimately contacts S6 for virtually its entire length, forming a molecular zipper (Deng et al., 2018). As a result, gating stimuli acting on S1-S4 may exert force on S6 through S3 to open the channel gate (Deng et al., 2018). In contrast to other TRPV channels, the ion conduction pore of TRPV4 contains only one intracellular gate, with the narrowest region at residue M714 (Deng et al., 2018). The selectivity filter of TRPV4 is also remarkably wide, allowing the conduction of Na^+^, K^+^ and Ca^2+^ even when hydrated (Deng et al., 2018). Thus, TRPV4 is a nonselective cation channel with higher permeability for divalent ions, such as Ca^2+^, Ba^2+^ and Mg^2+^, than monovalent ions, such as Na^+^, Cs^+^ and K^+^ (Voets et al., 2002). Endogenous TRPV4 agonists include arachidonic acid, endocannabinoid anandamide, epoxyeicosatrienoic acids and phorbol derivatives (Clapham, 2003). Additionally, PIP_2_ regulates TRPV4 sensitivity to temperature and hypotonicity through a phosphoinositide-binding site,^121^KRWRK^125^ (Garcia-Elias et al., 2013). TRPV4 can also be inhibited or potentiated in a Ca^2+^ concentration-dependent manner and function as an inwards or outward rectifier (Voets et al., 2002)

Compressive forces in the primary tumor, fluid shear stress in the blood vessels, and other mechanical stimuli that cause local changes in cell membrane curvature, composition and tension can directly open MICs such as Piezo1/2, transient receptor potential cation channel subfamily C member 1 (TRPC1), and transient receptor potential cation channel subfamily V member 4 (TRPV4) (Petho et al., 2019). Alternatively, mechanosensitive molecules on the plasma membrane can indirectly trigger MIC activation through a multi-step process involving several intermediate signaling molecules. This mechanism typically relies on the ability of G-protein coupled receptors to detect changes in the extracellular environment and induce a conformational change in MICs, such as transient receptor potential cation channel subfamily M member 7 (TRPM7) and TRPV4 (Petho et al., 2019). Self-generated forces arising from actomyosin contractility can also lead to the activation of MICs in migrating cells (Lee et al., 2021). For example, tension in actin cytoskeletal scaffolds and enhanced signaling through integrins (cell-matrix interactions) and cadherins (cell-cell interactions) can indirectly influence the gating of MICs (Petho et al., 2019).

While the role of MICs in nerves and sensory cells involved in sight, smell, taste and touch is well-characterized (Ranade et al., 2015), their expression and function in cancer progression has recently received attention (Adapala et al., 2016; Dombroski et al., 2021; Karki and Tojkander, 2021; Yankaskas et al., 2021). Given that cancer cells are subject to a multitude of forces that continuously change with the local microenvironment, it is important to understand how MICs regulate cell behavior during each step of the metastatic cascade. Here, we provide a comprehensive overview of physical cues that cancer cells experience and how MICs allow cells to sense and interpret these forces. In particular, we discuss the roles of Piezo1/2, TRPC1, TRPC5, TRPM2, TRPM4, TRPM7, TRPV2, and TRPV4 in mechanosensing and cancer metastasis.

### Piezo1/2 Mechanosensitive Ion Channels

Piezo1 (Fam38A) and Piezo2 (Fam38B) are mechanically-activated cation channels located in the plasma membrane of vertebrates (Coste et al., 2010). Structurally, they are pore-forming membrane proteins with numerous transmembrane regions that are directly activated by mechanical stimuli applied to the cell membrane like other MICs (Table 2); however, they do not share sequence homology with any known ion channels or receptors. Compared to other MICs, which can be activated by both mechanical and chemical stimuli, Piezo1/2 are the only channels primarily gated by mechanical stimuli (De Felice and Alaimo, 2020), including compression, shear stress, membrane stretching, poking, and suction (Table 1).

Since their discovery in 2010, the physiological roles of Piezo channels have been extensively studied. Piezo1 was initially identified in mouse neuroblastoma cells as a critical mediator of mechanically-activated currents, but subsequently found to be expressed in the bladder, colon, lungs, kidneys and skin (Coste et al., 2010). Piezo1 plays essential regulatory roles in bone anabolism (Li et al., 2019a), iron metabolism (Ma et al., 2021), innate immunity (Solis et al., 2019), vascular development (Ranade et al., 2014a) and erythrocyte volume homeostasis (Cahalan et al., 2015; Cinar et al., 2015). Piezo1 is also required for neurogenesis (Pathak et al., 2014), cell migration (Hung et al., 2016; Mousawi et al., 2020) and axonal pathfinding and growth (Koser et al., 2016). Additionally, Piezo1 controls epithelial cell numbers by inducing cell division in sparse environments and cell extrusion in crowded environments (Gudipaty et al., 2017), and regulates endosomal trafficking for efficient cytokinetic abscission of nascent daughter cells (Carrillo-Garcia et al., 2021). Consequently, Piezo1 expression is required for embryonic development (Ranade et al., 2014a).

Piezo2, which shares ∼42% sequence homology with Piezo1, was identified in dorsal root and trigeminal ganglia sensory neurons, where it facilitates a subset of kinetically distinct mechanically-activated currents involved in somatosensation and proprioception (Coste et al., 2010; Chesler et al., 2016). Piezo2 is also expressed in the bladder, colon, lungs, kidneys and skin (Coste et al., 2010). Like Piezo1, Piezo2 participates in a diverse set of mechanisms, including Merkel cell sensitivity to light touch, itch, mechanical pain, proprioception, baroreflex, breathing and bladder control (Szczot et al., 2021). Piezo2 is primarily involved in mechanosensation; however, global knockout of gene expression results in perinatal lethality (Ranade et al., 2014b) like Piezo1 (Ranade et al., 2014a). Thus, there are likely many other functions of Piezo2 that remain to be identified.

A growing body of evidence indicates that the expression and function of Piezo1/2 are altered in cancer. Piezo1 is abnormally expressed in tissues typically exposed to high levels of mechanical stress. Specifically, breast (Li et al., 2015), colorectal (Sun et al., 2020), gastric (Wang et al., 2021) and prostate (Han et al., 2019) cancer exhibit elevated levels of expression, as well as gliomas (Zhou et al., 2020) and oral squamous cell carcinoma (OSCC) (Hasegawa et al., 2021). Piezo2 is also upregulated in breast cancer (Pardo-Pastor et al., 2018) and gliomas (Yang et al., 2016).

### Mechanosensitive transient receptor potential channels

The TRP superfamily of MICs consists of 7 different subfamilies based on sequence homology, including TRPA, TRPC, TRPM, TRPML, TRPN, TRPP, and TRPV, all of which are expressed in mammalian cells except for TRPN (Montell, 2005). TRP channels are primarily localized to the plasma membrane and endoplasmic reticulum and can be activated by various stimuli such as heat, tension, pH, osmolarity and pressure (Table 1) (Pedersen and Nilius, 2007; Patel et al., 2010). Most TRP channels function as tetramers with six putative transmembrane segments (S1-S6) and a pore-forming re-entrant loop between S5 and S6. Except for TRPM4 and TRPM5, most TRPs are also non-selective cation channels that permit Na^+^, K^+^, Ca^2+^ and/or Mg^2+^ influx to varying degrees depending on their specific structure. Increased cation entry can affect cellular function directly or by enhancing intracellular signaling cascades which lead to the downstream release of calcium (Pedersen and Nilius, 2007; Petho et al., 2019). Importantly, TRP channels also regulate the proliferation, differentiation, migration, invasion and chemoresistance of cancer cells by sensing osmotic perturbations, shear forces and hydrostatic pressure (Clapham, 2003; Liedtke et al., 2003; Gomis et al., 2008; Mendoza et al., 2010; Shen et al., 2015; Zhao et al., 2019; Yankaskas et al., 2021; Zhao et al., 2021) (Table 1). Several members of the TRP superfamily also participate in actin remodeling and focal adhesion dynamics in response to mechanical stimuli (Kobayashi and Sokabe, 2010; Shen et al., 2015; Zhao et al., 2019). TRP channels implicated in mechanotransduction mechanisms that regulate cancer cell migration, invasion and metastasis include, among others, TRPC1, TRPC5, TRPM2, TRPM7, TRPM4, TRPV2 and TRPV4 (Table 3) (Patel et al., 2010; Canales et al., 2019).

**TABLE 3:** Ion channels involved in tuning cell behavior in response to physical cues.

Ion channel	Physical cue	Roles
Piezo1	Confinement (Hung et al., 2016)	Cell motility (Yang et al., 2014; Hung et al., 2016; Zhang et al., 2018; Han et al., 2019)
	Mechanical perturbations (Coste et al., 2012; Ranade et al., 2015; Woo et al., 2015; Cox et al., 2016)	Cell proliferation (Zhang et al., 2018; Han et al., 2019; Sun et al., 2020; Hasegawa et al., 2021; Wang et al., 2021)
	Voltage (Moroni et al., 2018)	Invasion (McHugh et al., 2012; Li et al., 2015; Etem et al., 2018; Huang et al., 2019; Yu et al., 2021)
Piezo2	Narrow pores (Pardo-Pastor et al., 2018)	Cell proliferation (Yang et al., 2016; Etem et al., 2018)
	Mechanical perturbations (Coste et al., 2012; Ranade et al., 2015; Woo et al., 2015; Cox et al., 2016)	Invasion (McHugh et al., 2012; Yang et al., 2016; Jiang et al., 2017; Etem et al., 2018; Pardo-Pastor et al., 2018; Huang et al., 2019)
	Voltage (Moroni et al., 2018)	Cell motility (McHugh et al., 2012; Yang et al., 2016; Etem et al., 2018; Pardo-Pastor et al., 2018; Huang et al., 2019)
TRPC1	Pressure (Li et al., 2019c)	Cell proliferation (El Hiani et al., 2009; Sobradillo et al., 2014; Faouzi et al., 2016; Zhang et al., 2020; Sun et al., 2021)
		Cell motility (Fabian et al., 2008; Dong et al., 2010; Zhang et al., 2020; Sun et al., 2021)
		Invasion (He et al., 2012; Sobradillo et al., 2014)
TRPM2	Oxidative stress (Perraud et al., 2005; Huang et al., 2018)	Cell motility (Li et al., 2016; Lin et al., 2018; Almasi et al., 2019; Lin et al., 2021)
		Cell proliferation (Almasi et al., 2018; Lin et al., 2018; Lin et al., 2021)
		Invasion (Lin et al., 2018; Almasi et al., 2019; Lin et al., 2021)
TRPM4	Membrane stretch (Morita et al., 2007)	Cell proliferation (Kappel et al., 2019)
		Cell motility (Cáceres et al., 2015; Kappel et al., 2019; Sagredo et al., 2019)
		Invasion (Cáceres et al., 2015; Kappel et al., 2019; Sagredo et al., 2019)
TRPM7	Shear stress (Yankaskas et al., 2021) Hydraulic resistance (Zhao et al., 2019; Zhao et al., 2021)	Intravasation (Yankaskas et al., 2021)
		Track choice during cell migration (Zhao et al., 2019) and morphology (Zhao et al., 2021)
		Cell proliferation (Sun et al., 2014a; Wang et al., 2014; Gao et al., 2017; Liu et al., 2019b; Su et al., 2019)
		Cell motility (Chen et al., 2010; Middelbeek et al., 2012; Meng et al., 2013; Sun et al., 2014a; Wang et al., 2014; Gao et al., 2017; Liu et al., 2019b; Su et al., 2019; Yang et al., 2020a; Lefebvre et al., 2020)
		Invasion (Meng et al., 2013; Wang et al., 2014; Gao et al., 2017; Liu et al., 2019b; Su et al., 2019; Yang et al., 2020a)
		Apoptosis (Su et al., 2019)
TRPV2	Membrane stretch (Mihara et al., 2010; Shibasaki et al., 2010)	Cell proliferation (Kudou et al., 2019)
		Cell motility (Liu and Wang, 2013; Kudou et al., 2019; Kato et al., 2022)
		Invasion (Liu and Wang, 2013; Kudou et al., 2019; Kato et al., 2022)
		Apoptosis (Kudou et al., 2019)
TRPV4	Stiffness (Adapala et al., 2016; Cappelli et al., 2019; Sharma et al., 2019)	Cell motility (Mrkonjić et al., 2015; Lee et al., 2017a; Ou-Yang et al., 2018; Li et al., 2020c; Zhang et al., 2021)
		Invasion (Ou-Yang et al., 2018; Yang et al., 2020b; Zhang et al., 2021)
		Extravasation (Lee et al., 2016)

### Physical cues encountered at the primary tumor site

Cancer arises from defects in regulatory circuits that control normal cell processes. Several lines of evidence suggest that tumor cells acquire a self-sufficiency in growth signals, an insensitivity to growth suppression, limitless replicative potential, sustained angiogenesis and the ability to evade apoptosis (Hanahan and Weinberg, 2011). These characteristics enable tumor cells at the primary site to outgrow and dominate their local tissue environment.

Cells encounter a variety of physical forces within the primary tumor, including mechanical compression, hydraulic pressure and fluid viscosity (Figure 1; Table 1). As described earlier, MICs are a diverse group of ion channels that allow cells to sense, interpret and respond to a multitude of mechanical stimuli (Ranade et al., 2015). Thus, a detailed understanding of these forces is required to contextualize how MICs may be involved in the first few steps of the tumor cell transformation and the initiation of cancer metastasis.

#### Compressive and radial stresses

As murine or human cancer cells proliferate in the mammary fat pad of mice, the total tumor volume expands and deforms the surrounding tissue, leading to the accumulation of compressive stress upwards of 150 mm Hg (20 kPa) (Stylianopoulos et al., 2012; Jain et al., 2014). Increasing tumor volume also generates mechanical compression in the tumor (Figure 1; Table 1). The accumulation of compressive stress is evident from force balance and the observed stress relaxation of tumor halves when excised tumors are cut along their longest axis; without the application of any external load, “cutting” releases internal stress, resulting in simultaneous bulging at the center and retraction at the boundary of the tumor, which are indicative of compression in the intratumoral region and radial stress at the periphery, respectively (Stylianopoulos et al., 2012).

Compressive stress in the tumor interior can influence the shape of the tumor mass and alter cancer cell biology, resulting in enhanced invasiveness (Nia et al., 2020). Stress levels equivalent to those encountered in the native breast tumor microenvironment drive cancer cells towards a more invasive phenotype (Tse et al., 2012) by altering gene expression profiles that remodel the ECM and tumor vessels (Demou, 2010; Rivron et al., 2012), thereby facilitating tumor dissemination out of a primary tumor mass. For example, compression can enhance breast cancer cell migration by inducing the formation of new adhesion contacts with the substrate *via* localized fibronectin secretion (Tse et al., 2012), and increase invasion by engaging matrix metalloproteinases (MMPs) (Luo et al., 2022). In particular, mechanical compression of breast cancer cells stimulates Ca^2+^ influx through Piezo1, leading to Src/ERK pathway activation, invadopodia formation, increased matrix degradation and enhanced cell invasion (Luo et al., 2022). The localization and function of Piezo1 is dependent on the integrity of caveolae in the cell membrane as knockdown of Cav-1 abrogates Ca^2+^ influx and cell invasion (Luo et al., 2022). High compressive stress inside spheroid models of breast cancer also promotes an invasive phenotype by facilitating water flow across cell boundaries from the tumor core to the periphery and invasive front *via* gap junctions (Han et al., 2020). Interestingly, this pattern of cell volume regulation is preserved in tissue samples from human patients; namely, cells positioned progressively further from the tumor core exhibit greater volumes (Han et al., 2020). Of note, high stress can induce growth arrest and apoptosis of murine mammary carcinoma cells in the interior of tumor spheroids, while anisotropic forces can pattern tumor volume increase in the direction of least stress (Cheng et al., 2009).

#### Hydraulic pressure

The hydraulic environment around tumors is largely determined by the composition of fluids combined with the structure and organization of blood and lymphatic vessels (Koumoutsakos et al., 2013). Dynamic ECM deformation enhances microvascular network formation *in vitro* by altering gene expression (Ruehle et al., 2020). Compressive stresses can also pinch blood and lymphatic vessels at the tumor interior and transform the cross-sectional area of such vessels at the peritumoral region into elliptical shapes. The compression of leaky blood vessels, which are frequently associated with cancer progression (Hashizume et al., 2000), results in elevated interstitial fluid pressure (IFP) (Table 1) (Cheng et al., 2009) by increasing fluid flux from the vasculature into interstitial spaces (Figure 1). Concurrently, compression of lymphatic vessels results in improper drainage of excessive interstitial fluids, which further increases IFP (Jain and Baxter, 1988; Baxter and Jain, 1989). Overall, the haphazard and leaky tumor vasculature contributes to higher IFP, which can drive local breast cancer cell dissemination and metastasis (Haessler et al., 2012; Li et al., 2020a).

Elevated IFP at the tip of three-dimensional (3D) aggregates of human breast cancer (MDA-MB-231) cells increases invasion by enhancing the expression of genes associated with epithelial-to-mesenchymal transition (EMT), namely Snail and vimentin (Piotrowski-Daspit et al., 2016). Interestingly, increased IFP at the invasive tips also enhances the expression of the epithelial marker E-cadherin (Piotrowski-Daspit et al., 2016), whose role in breast cancer progression is debated, yet has been demonstrated to support metastasis (Kowalski et al., 2003; Padmanaban et al., 2019). Elevated IFP also increases the volume, motility and invasiveness of human lung cancer (CL1-5 and A549) cells due to ERK1/2 pathway activation (Kao et al., 2017). Hydrostatic pressure initiates a cascade of caveolin-1, Akt1/2, ERK1/2 and cortactin phosphorylation which enhances filopodia formation and drives the increased expression of aquaporin 1 (AQP1), Snail and vinculin (Kao et al., 2017). Importantly, hydrostatic pressure does not significantly enhance the volume, motility or proliferation of normal lung epithelial (BEAS-2B) cells due to negligible changes in the expression of AQP1 and the phosphorylation status of caveolin-1 and ERK1/2 (Kao et al., 2017).

#### Extracellular fluid viscosity

The viscosity of extracellular fluids also constitutes an important physical parameter that varies throughout the human body during both healthy and diseased states (Figure 1; Table 1). The fluids surrounding primary tumors have elevated viscosity due to deregulated blood circulation (Sun et al., 2007) and increased amounts of ECM degradation (Ellis, 2001). Additionally, resident epithelial and cancer cells secrete macromolecules, such as mucins, which further increase fluid viscosity (Ellis, 2001). Magnetic resonance elastography studies of patient samples reveal local upregulation of shear viscosity at tumor sites, with malignant breast tumor masses displaying 3-fold greater shear viscosity than benign tumors (Sinkus et al., 2005; Kumar et al., 2018). Although magnetic resonance electrograms measure the material properties of bulk tumor masses, a recent development in molecular rotor-based fluorescent sensors has enabled the measurement of fluid viscosities *in vivo* (Yin et al., 2021). Importantly, *in vivo* imaging of a viscosity-activated fluorescent probe in mice bearing murine breast tumors confirmed the existence of significantly elevated fluid viscosity in tumors compared to native tissues (Yin et al., 2021). Unfortunately, it remains elusive how cells in the primary tumor respond to physiologically-relevant values of viscosities. Further work is required to elucidate the impact of extracellular fluid viscosity on cancer cell behavior and uncover the molecular mechanisms that are engaged by this physical cue.

### Mechanical properties and geometrical constraints during cell invasion into the local microenvironment

ECM structure surrounding primary tumor sites is often modified compared to native tissues (Winkler et al., 2020) resulting in unique topographies which cells can use to invade out of the primary tumor region. Cancer cells can remodel the ECM by promoting the secretion and deposition of additional matrix components; altering the organization of fibers through proteolytic cleavage and chemical modifications; and applying mechanical forces on existing fibers (Winkler et al., 2020). In turn, cells can sense and respond to material properties and the architecture of ECM fibers (Sharma et al., 2013; Doyle et al., 2015; Mukherjee et al., 2019). Increased collagen deposition, alignment and cross-linking affect the stiffness (Micalet et al., 2021) and viscoelasticity (Mierke, 2021) of the local microenvironment and establish migration paths for cells to escape the primary tumor (Figure 1).

#### Substrate stiffness

ECM deposition, modification and organization during cancer progression contribute to increased matrix stiffness or elasticity (Figure 1; Table 1). In the case of breast cancer, shear wave elastography reveals that tumor stiffness increases with tumor grade and disease progression; benign lesions measure ∼45 ± 40 kPa whereas malignant lesions can reach ∼147 ± 40 kPa (Athanasiou et al., 2010; Bae et al., 2017). Nanoscale atomic force microscopy indentation of breast tissue samples also demonstrates a dramatic stiffening of invasive ductal carcinomas (>5 kPa) compared to normal tissue (∼0.4 kPa) (Paszek et al., 2005). Additionally, stromal stiffness at the invasive edge of tumors is 4-fold greater than non-invasive regions (Paszek et al., 2005).

Collagen is the most abundant ECM component deposited during breast cancer progression (Schedin and Keely, 2011; Naba et al., 2014). Increased stromal collagen in mice promotes tumor initiation, cancer cell invasion and lung metastasis (Provenzano et al., 2008). Accordingly, elevated collagen is a risk factor for breast cancer (Li et al., 2005). Increased collagen crosslinking and stiffening of the ECM promote focal adhesion formation and enhanced phosphoinositide 3-kinase (PI3K) activity in human mammary epithelial cells. Along these lines, inhibition of lysyl oxidase (LOX)-mediated collagen crosslinking decreases focal adhesions, reduces PI3K activity, and impedes tumor progression in mice (Levental et al., 2009). Deregulated ECM architecture also affects the progression of many other cancer types, including colon (Birk et al., 2014), prostate (Kapinas et al., 2012) and ovarian (Nadiarnykh et al., 2010) cancer. It is important to note that the stiffness of the local microenvironment varies considerably due to tumor heterogeneity (Plodinec et al., 2012). Tissue stiffness also varies widely throughout the body (Handorf et al., 2015). Thus, cancer cells must constantly sense and adapt to local changes in stiffness as they disseminate from the primary tumor and migrate towards the vasculature (Figure 1).

Cells can sense the rigidity and topography (Table 1) of the underlying ECM through cell-matrix adhesions (Box 1) (Geiger et al., 2009), which connect the cellular cytoskeleton to the local microenvironment. More recently, the role of MICs in substrate stiffness sensing has also emerged. For example, primary mouse chondrocytes cultured on polydimethylsiloxane (PDMS) exhibit elevated Ca^2+^ signaling with increasing stiffness (2–197 kPa); TRPV4 preferentially mediates Ca^2+^ influx on stiffer (197 and 78 kPa) substrates, whereas Piezo1/2 facilitates Ca^2+^ influx on softer (54 and 2 kPa) substrates (Du et al., 2021). In the context of prostate cancer, TRPV4 expression in murine tumor endothelial cells regulates Ca^2+^ influx and Rho activity in response to substrate stiffness (Adapala et al., 2016). Endothelial cells with diminished levels of TRPV4 exhibit reduced mechanosensitivity, which increases cell migration on stiffer substrates and decreases VE-cadherin at cell-cell junctions, leading to abnormal angiogenesis, enhanced tumor growth, and enhanced lung metastasis (Adapala et al., 2016; Cappelli et al., 2019).

Box 1 | **Cell-matrix adhesions are important structures that facilitate cancer cell migration and mechanosensing. Adhesions are composed of integrins and cytoplasmic proteins that form a <200 nm plaque containing at least 156 components with 690 interactions (Zaidel-Bar et al., 2007a; Kuo et al., 2011). There are four main classes of adhesions which are distinguished by their size, protein composition and lifetime: nascent adhesions, focal complexes, focal adhesions and fibrillar adhesions (Wolfenson et al., 2013). Nascent adhesions at the leading edge of migratory cells are <1 μm in diameter, primarily composed of integrins, talin and paxillin, and have a lifespan <60 s (Laukaitis et al., 2001; Zaidel-Bar et al., 2003; Wolfenson et al., 2013). Nascent adhesions mature into focal complexes upon vinculin recruitment and attachment to the actin cytoskeleton (DePasquale and Izzard, 1987). Mechanical force promotes the recruitment of more scaffold proteins and strengthening of the adhesion-actin cytoskeleton link, leading to the formation of focal adhesions (Galbraith et al., 2002; Choi et al., 2008). Focal adhesions are larger than focal complexes (1 μm wide × 3–5 μm long), contain zyxin, and have a considerably longer lifetime (>8.5 min) (Zaidel-Bar et al., 2003; Zaidel-Bar et al., 2007b). Continued force application transitions focal adhesions into fibrillar adhesions, which are significantly longer, contain tensin, and have a very long lifetime (∼42 min) (Pankov et al., 2000; Zaidel-Bar et al., 2007b). Cells typically form adhesions along ECM bundles of collagen and fibronectin (Harunaga and Yamada, 2011; Doyle et al., 2015). Nascent adhesions are initially formed at the leading edge of cells in a force- and stiffness-independent fashion (Nayal et al., 2006; Choi et al., 2008). Many of these small adhesions quickly decay while a few continue to increase in size and move towards the cell interior (Parsons et al., 2010). Substrate stiffness increases the fraction of adhesions that mature into large complexes (Discher et al., 2005) by unmasking additional binding sites on adhesion proteins (Horton et al., 2016; del Rio et al., 2009). Adhesions on stiff matrices also experience stronger forces from actomyosin contraction, and generate stronger frictional forces on the actin cytoskeleton (Walcott and Sun, 2010). For this reason, collagen crosslinking and matrix stiffening promote adhesion formation and maturation (Levental et al., 2009; Provenzano et al., 2009; Walcott et al., 2011). Increased ECM stiffness also induces EMT (Wei et al., 2015), which is associated with tumor cell dissemination, immune evasion and chemoresistance (Aiello and Kang, 2019). Mesenchymal cell migration is highly dependent on adhesion dynamics. Accordingly, upregulation of proteins that enhance actin cytoskeleton and adhesion dynamics is often observed in invasive and metastatic cancer cells, while inhibition of these mediators is beneficial in blocking cell migration (Yamaguchi and Condeelis, 2007; Wendt and Schiemann, 2009). Force fluctuations within adhesions are converted into biochemical signals through a focal adhesion kinase (FAK)/phosphopaxillin/vinculin signaling pathway (Plotnikov et al., 2012). Other studies have also identified FAK (Klein et al., 2009; Wang et al., 2019), vinculin (Plotnikov et al., 2012; Stutchbury et al., 2017), talin (del Rio et al., 2009; Austen et al., 2015; Elosegui-Artola et al., 2016) and zyxin (Yoshigi et al., 2005; Hirata et al., 2008) as mechanosensitive proteins required for substrate stiffness sensing. These proteins allow cells to rapidly respond to changes in substrate stiffness (Pelham and Wang, 1997; Lo et al., 2000) and migrate towards stiffer areas almost immediately after substrate stretching or compression (Lo et al., 2000) through a process known as durotaxis (DuChez et al., 2019). Mathematical models of steady-state cell speed suggest cells migrate optimally within a narrow range of stiffnesses, with soft or very stiff substrates both resulting in less cell migration (Pelham and Wang, 1997; Dokukina and Gracheva, 2010). In agreement with this hypothesis, glioma (U251) cells exhibit preferential migration towards substrates with a stiffness of 10 kPa from both softer and stiffer regions (Bangasser et al., 2017). Small adhesions formed on soft substrates fail to support traction forces required for cell migration. Conversely, stiffer substrates induce cell spreading, adhesion formation, and adhesion maturation, which collectively result in slower migration (Discher et al., 2005).**

It has been postulated that local stretching of the plasma membrane near focal adhesions (Box 1) activates MICs, which ultimately control cell migration by regulating actomyosin contractility and the fate of focal adhesions (Kobayashi and Sokabe, 2010). Ca^2+^ flickers have been observed near adhesions in migrating human embryonic lung fibroblasts (Wei et al., 2009). Rapid local application of an RGD sequence recognized by integrins enhances flicker activity. In contrast, TRPM7 knockdown and myosin inhibition prevent flicker production (Wei et al., 2009). Ca^2+^ entry through membrane-associated channels impacts adhesion dynamics by activating calpain, a cysteine protease (Khorchid and Ikura, 2002) that cleaves several adhesion components, including integrin (Du et al., 1995), focal adhesion kinase (FAK) (Chan et al., 2010) and talin (Franco et al., 2004). In fact, calpain-mediated proteolysis of talin is a rate-limiting step during adhesion turnover (Franco et al., 2004); calpain inhibition decreases integrin release from the cell membrane, leading to a reduction in cell speed (Huttenlocher et al., 1997). Accordingly, human glioma (U87), breast cancer (T47D) and embryonic kidney (HEK) cells expressing a defective form of TRPV4 exhibit long protrusions with large, elongated adhesions that prevent migration (Mrkonjić et al., 2015). In contrast, cells overexpressing wildtype TRPV4 possess very few and small adhesions (Mrkonjić et al., 2015). TRPV4 inhibition in human mesenchymal stem cells also decreases tensile forces across vinculin and dramatically blocks collagen fibril assembly *in vitro* (Gilchrist et al., 2019). Moreover, TRPV4 activation in human breast cancer (4T07) cells enhances Akt and FAK phosphorylation and transendothelial migration (Lee et al., 2017a). Piezo1 has also been shown to enhance the growth of human glioblastoma stem cells and promote tumor development by activating integrin/FAK signaling (Chen et al., 2018). Piezo1 knockdown cells fail to assemble focal adhesion structures which halts a positive feedback loop that otherwise allows glioma cells to remodel the local ECM, increase tissue stiffness, and further enhance Piezo1 activity (Chen et al., 2018). As such, doxycycline-mediated shRNA knockdown of Piezo1 suppresses tumor growth and prolongs the survival of mice (Chen et al., 2018). Interestingly, TRPV4 expression in normal mouse primary epidermal keratinocytes has been shown to regulate transforming growth factor β (TGFβ)-induced EMT and nuclear translocation of Transcriptional coactivator with PDZ-binding motif (TAZ) in response to matrix stiffness (Sharma et al., 2019); however, these findings remain to be investigated in cancerous cells.

#### Viscoelasticity

While stiffness is an important prognostic factor that enhances metastatic phenotypes, it is important to note that most biological tissues and ECMs are not purely elastic (Chaudhuri et al., 2020). Tissues exhibit a time-dependent mechanical response and dissipate a fraction of energy it took to deform them, a property known as viscoelasticity (Table 1). Soft tissues, such as brain, liver and lung, generally exhibit viscous moduli that are 10%–20% of their elastic moduli (Chaudhuri et al., 2020). Even hard tissues, such as bone and cartilage, display viscoelastic behaviors with viscous moduli that are about 10% of their elastic moduli (Chaudhuri et al., 2020). Thus, most biological tissues undergo permanent deformation following the application of external force. Importantly, magnetic resonance elastography (Sinkus et al., 2007) and ultrasonic strain imaging (Bayat et al., 2018) and elastography (Kumar et al., 2018) reveal that malignant lesions are more viscoelastic than benign lesions.

Malignant tumors have increased collagen density (Li et al., 2005) with collagen fibers that are structurally different from those of normal ECM stroma (Provenzano et al., 2006). Elevated collagen concentrations, the elongation of individual fibers, and the formation of new crosslinks substantially increase plasticity (Figure 1) (Ban et al., 2018). Viscoelastic 2D substrates enhance the migration of human fibrosarcoma (HT-1080), breast carcinoma (MDA-MB-231), and mammary epithelial cells by promoting the formation of filopodial protrusions and nascent adhesions; fascin1, formin and myosin-X facilitate filopodia formation, while integrin *β*_1_, Arp2/3, Rac1 and myosin participate in adhesion-based motility (Adebowale et al., 2021). Importantly, MDA-MB-231 cells in viscoelastic 3D matrices with a stiffness of ∼1.8 kPa can extend invadopodia and mechanically enlarge the gel pores to enhance migration through the ECM (Wisdom et al., 2018). Human chondrocytes are able to sense changes in substrate viscoelasticity *via* TRPV4, which in turn regulates the phosphorylation of glycogen synthase kinase 3β (GSK3β) (Agarwal et al., 2021). However, the molecular mechanisms through which cancer cells sense substrate viscoelasticity remain unknown and the effect of viscoelasticity on cancer cell metastasis requires further investigation. Nevertheless, these results implicate viscoelasticity as an important physical cue that affects cell migration and invasion.

#### Contact guidance along topographical features

Cells can align to nano- and micro-scale topographical features of the substrate and migrate along them using contact guidance (Table 1) (Martinez et al., 2009; Paul et al., 2016). Second harmonic generation (SHG) imaging of human breast cancer biopsies and mouse tumors shows that collagen fibers are increasingly aligned in invasive versus benign stages of cancer and in more aggressive tumors (Provenzano et al., 2006; Conklin et al., 2011). Intravital imaging also demonstrates that breast cancer cell invasion out of the primary tumor is predominantly oriented along aligned collagen fibers (Condeelis and Segall, 2003; Provenzano et al., 2006). Remarkably, primary tumor explants from mice cultured in a randomly organized collagen matrix can realign collagen fibers to promote outward migration of individual cancer cells along radially oriented fibers (Provenzano et al., 2006). Consequently, collagen fiber alignment relative to the breast tumor interface is an independent prognostic marker for disease progression (Li et al., 2005; Zunder et al., 2020) and survival (Paul et al., 2016).

Cancerous and non-cancerous cells can wrap around suspended fibers that are 0.1–1 µm in diameter and alter their protrusion dynamics depending upon the fiber curvature (Mukherjee et al., 2019). For example, human glioblastoma (U251) cells seeded on suspended nanofibers migrate faster and more persistently on 1D geometries than on 2D orthogonal fibers, and cells on closely spaced parallel fibers achieve even faster speeds than on single fibers (Estabridis et al., 2018). These three migratory behaviors are successfully recapitulated by a mathematical model that uses motor-clutch based force transmission (Chan and Odde, 2008), highlighting the role of topographical cues during glioblastoma cell migration (Estabridis et al., 2018). The stiffness and length of fibers also affects the migration speed and phenotype of human glioma (DBTRG-05MG) cells (Sharma et al., 2013). Motile cells can also align parallel to ridges on a 2D surface. Interestingly, using laminin-coated ridges with sub-micron features, the migratory phenotype of 14 patient-derived glioblastoma samples in response to platelet-derived growth factor (PDGF) could predict the tumor recurrence in the clinic (Smith et al., 2016).

Besides the direct sensing of native topographical cues by cancer cells, cancer associated fibroblasts (CAFs), which are abundantly present in the tumor microenvironment, alter the ECM architecture by producing collagen, fibronectin, and many other matrix components (Winkler et al., 2020). CAFs can also secrete LOX (Karagiannis et al., 2012; Liu et al., 2019a) and interact with collagen-rich ECM through focal adhesions to align fibers *via* RhoA-mediated activation of myosin light chain activity (Gaggioli et al., 2007; Goetz et al., 2011). Taken together, deregulated ECM architecture provides distinct topographical cues that facilitate tumor cell invasion into the surrounding tissue. Thus, contact guidance along ECM fibers and topographical features act as clinically relevant physical cue of cancer progression.

#### Confined tracks and microchannels

The ECM contains pores of varying sizes from 1 to 20 µm in diameter and narrow tube-like tracks that are 3–30 µm in width and up to 600 µm long, due to the organization and cross-linking of matrix components (Wolf et al., 2009). Cancer cells can physically widen or enzymatically degrade the surrounding ECM to create their own migration tracks (Bremer et al., 2001; Fisher et al., 2009; Wisdom et al., 2018), follow paths created by other cancer cells and CAFs (Gaggioli et al., 2007; Patsialou et al., 2013), or move through pre-existing channel-like tracks present in the native environment (Friedl and Alexander, 2011; Weigelin et al., 2012) (Figure 1).

Cells typically adapt to the local environment and migrate through the ECM by selecting the path of least resistance (Renkawitz et al., 2019; Yamada and Sixt, 2019; Zhao et al., 2019). To migrate through small channels and openings efficiently, cells rely on mechanosensitive mechanisms that detect changes in confinement and modulate their migration strategy accordingly (Hung et al., 2013). Using microfabricated PDMS devices and substrate printing methods, it was shown that confinement increases Ca^2+^ influx in Chinese Hamster Ovary (CHO) cells *via* the stretch-activated cation channel Piezo1 to reduce the activity of cyclic AMP (cAMP)-dependent protein kinase A (PKA) near the plasma membrane (Hung et al., 2016). Similarly, Ca^2+^ influx through Piezo2 was shown to facilitate efficient migration of brain metastatic MDA-MB-231 cells through narrow channels by modulating the activity of RhoA and the formation and orientation of stress fibers and focal adhesions (Pardo-Pastor et al., 2018). One possible mechanism linking Piezo2 and RhoA activation involves Fyn kinase recruitment and calpain activation at the leading edge of migrating cells (Pardo-Pastor et al., 2018). The coordinated activity of these proteins is known to regulate focal adhesion dynamics and stress fiber formation (Pardo-Pastor et al., 2018).

Remarkably, cells can squeeze through confining pores narrower than their resting dimensions by distorting the shape of their cell body and organelles; however, as the largest and stiffest cellular component, the nucleus determines the smallest pore size cells can pass through without degrading the surrounding matrix (Friedl et al., 2011; Wolf et al., 2013). Indeed, MMP-independent migration linearly decreases with pore size until the nucleus deforms down to ∼10% of its original cross section, at which point cells are no longer able to traverse the physical constraint (7 μm^2^ for tumor cells, 4 μm^2^ for T cells and 2 μm^2^ for neutrophils) (Wolf et al., 2013). Cells with reduced lamin-A expression possess more malleable nuclei and migrate through pores more quickly; however, the chance of apoptosis is increased due to nuclear envelope (NE) rupture which promotes DNA damage (Harada et al., 2014; Denais et al., 2016; Raab et al., 2016; Mistriotis et al., 2019). DNA damage repair pathways are rapidly recruited following NE rupture to mitigate apoptosis; inhibiting their activation substantially increases cell death after nuclear rupture (Denais et al., 2016; Raab et al., 2016). Even in the absence of NE rupture, mechanical deformation of the nucleus is sufficient to cause DNA damage (Shah et al., 2021). Thus, there is a delicate balance between enhanced cell migration and DNA damage in confinement.

The nucleus can help cells probe paths of different cross-sectional area and direct cell entry into channels with the least resistance by serving as a mechanical gauge (Renkawitz et al., 2019). Cells can measure the degree of spatial confinement *via* Ca^2+^ release from internal stores. Vertical compression of the nucleus in human cervical cancer (HeLa-Kyoto) cells triggers unfolding of the inner nuclear membrane and the activation of cytosolic phospholipase A2 (cPLA2), which increases lipid arachidonic acid (ARA) production, Ca^2+^ release from internal stores, and myosin II activity (Lomakin et al., 2020; Venturini et al., 2020). Such mechanotransduction allowed cells to rapidly adapt (<1 min) their behavior to changing tissue environments, yet the responses were stable over time (>60 min) (Lomakin et al., 2020; Venturini et al., 2020). Given that MICs, such as Piezo1 (Gudipaty et al., 2017) can be found on the nuclear membrane, it will be interesting to investigate how the nucleus and Ca^2+^ permeable MICs potentially act as a comprehensive mechanosensory module.

#### Hydraulic pressure and resistance

As cancer cells migrate out of the primary tumor and begin to overcome the multitude of obstacles posed by the ECM, they are also subjected to a variety of physical forces imposed by the extracellular fluid (Figure 1). For example, cells uptake and discharge water during confined cell migration and/or displace a column of fluid ahead of them, which generates hydraulic resistance (Table 1) (Stroka et al., 2014a; Li et al., 2020a). Neutrophil-like cells are capable of sensing small changes in hydraulic pressure on the order of ∼1 Pa and display bias towards the path of lower hydraulic resistance when presented with multiple confining paths (Prentice-Mott et al., 2013). Similarly, cancer cells choose the path of least resistance during confined migration (Zhao et al., 2019). In particular, hydraulic pressure triggers Ca^2+^ influx through TRPM7 in HT1080 and MDA-MB-231 cells to generate a thick cortical actin meshwork with an elevated density of myosin-IIA, which directs cell entry into channels of less resistance (Zhao et al., 2019). CRISPR/Cas9 knockout of TRPM7 blinds cells to hydraulic resistance and causes them to choose migratory paths based on cross-sectional area (Zhao et al., 2019). TRPM7 not only influences the directional navigation of cancer cells but also provides cells with plasticity to counter elevated hydraulic resistances (Zhao et al., 2021). Elevated hydraulic resistance induces a shift in cell migration phenotype from amoeboid to mesenchymal (Zhao et al., 2021), which relies on the formation of actin-rich lamellipodia and integrin-based cell-matrix adhesions rather than membrane blebs (Friedl and Wolf, 2010; Pankova et al., 2010). This transition occurs *via* intricate modulation of actomyosin turnover machinery initiated by TRPM7-mediated calcium signaling (Zhao et al., 2021), and promotes faster cell migration through confinement (Wisniewski et al., 2020; Zhao et al., 2021). Elevated hydraulic resistance is not only encountered during cell migration through confined spaces with stiff channel walls; different ECM architectures can also influence the resistance faced by cells in 3D (Maity et al., 2019). In fact, collagen or ECM permeability can play a major role in dictating the hydraulic resistance experienced by a cell (Maity et al., 2019). Taken together, the hydraulic pressure and resistance of static fluids significantly influences the detachment of cancer cells from the primary tumor and the migratory path taken by cells as they traverse the ECM.

#### Extracellular fluid viscosity

Tumor sites possess increased fluid resistance and likely contain interstitial fluids with elevated viscosities (Jain et al., 2014); however, the contribution of these forces towards cancer cell dissemination and metastasis has largely been overlooked. Most cell migration studies to date have been exclusively performed using media which has a viscosity close to that of water (0.77 cP at 37°C). In contrast, the viscosity of interstitial fluid can reach 3.5 cP (Wells and Merrill, 1961; Yao et al., 2013; Gonzalez-Molina et al., 2018), while whole blood has a viscosity of 4–6 cP, which can be further elevated in pathological conditions (Rosenson et al., 1996). Several clinical studies demonstrate that fluid viscosity is correlated with negative outcomes in cancer patients. Elevated hematocrit levels increase the viscosity of whole blood which enhances metastasis in melanoma patients (Dintenfass, 1977). Similarly, high whole blood viscosity is associated with advanced stages of head and neck carcinoma (Khan et al., 1995). Increased whole blood viscosity is also associated with extrahepatic metastasis and reduced survival of patients with hepatocellular carcinoma (Han et al., 2021). Moreover, elevated plasma viscosity has been reported as an independent prognostic marker for the overall survival of breast cancer patients (Han et al., 2021). Thus, the role of physiologically relevant extracellular fluid viscosity in cancer cell migration and metastasis should be urgently investigated. Of note, supraphysiological viscosities (40–64.7 cP) induce increased motility and cell spread area in hepatic carcinoma cells (Gonzalez-Molina et al., 2018). However, it is unknown how cells sense and respond to fluid viscosities typically encountered *in vivo*.

### Fluid flow and shear stress encountered in the vasculature

Blood and lymphatic vessels in the human body provide a conduit for migrating tumor cells to travel throughout the body and colonize distant organs and tissues (Figure 1). To successfully form metastatic colonies, cancer cells must first intravasate through the endothelial lining of the bloodstream. *In vitro* studies reveal that MDA-MB-231 cells can create disruptions at least 20 µm in width to cross tissue-engineering microvessels (Wong and Searson, 2017). Disruption of endothelial cell-cell junctions is believed to be a key mediator of intravasation (Chiang et al., 2016); however, cancer cells can also enter the vasculature through pre-existing openings. For example, human breast cancer (MDA-MB-435) cells in zebrafish can intravasate into the bloodstream where it is actively being remodeled, but not at intact vessels (Stoletov et al., 2007). Murine mammary carcinoma models frequently display intercellular holes in tumor-associated vasculature (Hashizume et al., 2000). Tumor vasculature is predominantly leaky due to proteolytic degradation, remodeling/angiogenesis, paracrine signaling (such as the secretion of tumor necrosis factor (TNF)α) and hypoxia which disrupt the normal function of endothelial cells (Chiang et al., 2016). For this reason, intravasation typically occurs in the vicinity of the tumor where angiogenesis-induced capillary sprouts grow (Chiang et al., 2016). As mentioned previously, TRPV4 downregulation in murine endothelial cells also destabilizes tumor vessel integrity due to abnormal stiffness sensing and reduced VE-cadherin expression at cell-cell contacts (Adapala et al., 2016; Cappelli et al., 2019).

#### Fluid flow and shear stress during intravasation

Once cells enter the vasculature, they must withstand shear stress (Table 1) at the vessel wall (Figure 1). Due to the nature of fluid flow in conduits with a cylindrical cross section, shear stress is maximal at the blood vessel walls and ranges from 1 to 4 dyn/cm^2^ in venous circulation and 4–30 dyn/cm^2^ in arterial circulation (Turitto, 1982) with notable variations between different organs within the body. For example, turbulent blood flow (Table 1) in the heart can raise shear stress levels to as high as 1,000 dyn/cm^2^ (Moose et al., 2020). Shear stress is detrimental to the survival of cancer cells and leads to “metastatic inefficiency” (Weiss, 1990; Chiang et al., 2016). Using microfluidic devices that apply physiologically relevant levels of shear stress (0.5–5 dyn/cm^2^), it was recently shown that normal human dermal fibroblasts avoid exposure to regions of fluid flow, while HT-1080 cells readily enter such regions (Yankaskas et al., 2021). This finding is consistent with prior computational modeling showing that tumor cell intravasation predominantly occurs at sites of low shear stress (0.2–6 dyn/cm^2^) (Stapor et al., 2011). The behavior of normal fibroblasts is driven by elevated expression of TRPM7, which senses and activates RhoA-driven myosin-II contractility at the cell edge exposed to shear stress, and causes the reversal of migration direction *via* a calmodulin/IQGAP1/Cdc42 pathway (Yankaskas et al., 2021). As such, overexpression of TRPM7 in fibrosarcoma cells restores sensitivity to fluid shear stress, which markedly reduces their ability to intravasate *in vivo* (Yankaskas et al., 2021). Taken together, the integrity of the endothelial barrier and the level of shear stress generated by blood flow regulates the location and extent of intravasation.

#### Shear stress during circulation

Fluid flow in the circulatory system facilitates the transport of cancer cells throughout the body; however, cells must withstand shear stress for distant colonization (Table 1). Most circulating tumor cells (CTCs) that enter the circulation undergo apoptosis due to shear stress. Indeed, isolated CTCs shed from human colon adenocarcinoma (LS174T) cells and its highly metastatic subline (LS LiM 6) display a high degree of apoptosis compared to the native tumor population in mice (Swartz et al., 1999). Breast and prostate cancer patients also exhibit many apoptotic cells in their CTC population (Larson et al., 2004; Kallergi et al., 2013). Nevertheless, transformed cells are more resistant to fluid shear stress than their non-transformed counterparts (Barnes et al., 2012). Cancer cells can employ several strategies to cope with high variations in shear stress. For example, human prostate cancer (PC-3) cells become significantly stiffer upon exposure to shear stress compared to untransformed prostate epithelial (PrEC LH) cells, indicating a higher level of mechanical adaptability in cancer cells (Chivukula et al., 2015). PC-3, MDA-MB-231 and urinary bladder cancer (TCCSUP) cells can also activate RhoA signaling to protect themselves from shear-induced damage by restructuring the actin cytoskeleton using formin (Moose et al., 2020). Resistance to fluid shear stress is dependent on actomyosin contractility. Consequently, pre-treating PC-3 cells with myosin-II inhibitor delays metastasis formation in mice (Moose et al., 2020).

#### Shear stress during extravasation

Blood flow velocity can also affect the ability of CTCs to arrest and extravasate out of the vasculature (Table 1) by controlling the effectiveness and availability of ligand-mediated adhesion to the endothelial lumen. Blood flow velocities below 400–600 μm/s allow CTCs to arrest and adhere to endothelial cells in zebrafish embryos and mouse brain capillaries (Follain et al., 2018). Early arrest is mediated by weak adhesions composed of CD44 and integrins ɑ_v_ *β*_3_ which are subsequently stabilized by integrins ɑ_5_ *β*_1_ that bind fibronectin (Osmani et al., 2019). Of note, CD44 is a functional P-selectin ligand (Napier et al., 2007; Konstantopoulos and Thomas, 2009), which can also bind to E-selectin (and L-selectin) (Hanley et al., 2005; Hanley et al., 2006; Thomas et al., 2008), thereby mediating tumor cell tethering to endothelial cells under flow. Interestingly, a certain level of blood flow is required to promote the extravasation of CTCs, as reducing blood flow with lidocaine significantly decreases the percentage of cells that exit the vasculature in zebrafish embryos (Follain et al., 2018). This phenomenon is primarily driven by endothelial cells, which increase fibronectin deposition on their luminal surface in response to shear stress (Osmani et al., 2019). Similarly, *in vitro* studies using transendothelial assays show that hemodynamic shear stress increases the extravasation of MDA-MB-231 cells by inducing intracellular reactive oxygen species (ROS) production and ERK1/2 pathway activation (Ma et al., 2017). Accordingly, antioxidant suppression of ROS reduces tumor cell extravasation *in vitro* and in zebrafish (Ma et al., 2017). In a separate study, metastatic PC-3 cells were seeded on the luminal surface of PDMS tubes with a stiffness of 17 MPa and subjected to wall shear stress of 0.05 dyn/cm^2^ (Lee et al., 2017b) to mimic the stiffness of vascular walls and shear stress within interstitial or initial lymphatics (Swartz and Fleury, 2007; Resto et al., 2008). Fluid shear stress in this setting promoted an invasive phenotype characterized by filopodia (Lee et al., 2017b). Cells exposed to shear stress also migrated faster due to upregulated ROCK/LIMK/cofilin signaling, which induced Yes-associated protein (YAP)-mediated mechanotransduction and gene signature changes (Lee et al., 2017b). A recent study using a high-resolution humanoid blood flow model found that blood flow alone can predict to a high degree the distribution of metastatic site for a given primary tumor (Font-Clos et al., 2020). Thus, cells which can successfully withstand shear stresses found within the vasculature constitute a critical nucleator of secondary metastatic colonies.

### Mechanosensitive ion channel expression influences cancer progression

MICs play a key role in sensing various mechanical stimuli in the primary tumor, local microenvironment and the vasculature due to direct mechanosensing in many cancer cell types (Table 3). In addition to the mechanotransduction pathways described above, MICs have been reported to regulate numerous other intracellular biochemical pathways that influence cancer progression. While it is currently unknown how these pathways are linked to mechanical forces, it is evident that MICs play a diverse role in promoting metastatic phenotypes. In the following section, we provide an overview of how Piezo1/2, TRPC1, TRPC5, TRPM2, TRPM4, TRPM7, TRPV2 and TRPV4 affect cancer cell behavior. Channel characteristics and mechanisms of activation are summarized in Table 2.

#### Piezo1/2

Piezo1 overexpression is associated with poor outcomes in breast and colon cancer patients due to enhanced cell migration and invasion, respectively (Tables 2, 3) (Li et al., 2015; Sun et al., 2020). In particular, Piezo1 promotes the proliferation and migration of human colorectal cancer (SW-480 and HCT-116) cells *via* mitochondrial calcium uniporter (MCU), hypoxia-inducible factor (HIF)-1ɑ and vascular endothelial growth factor (VEGF) (Sun et al., 2020). Gastric cancer patients with Piezo1 upregulation also exhibit increased distant metastasis due to enhanced cell proliferation and motility (Yang et al., 2014; Zhang et al., 2018; Wang et al., 2021). Piezo1 enhances the migration of human gastric cancer cells by modulating the activity of Rho GTPase family members (Zhang et al., 2018), altering the expression of integrin subunits *via* Trefoil factor family 1 (TFF1) (Yang et al., 2014), and inducing the expression EMT-associated genes *via* HIF-1ɑ (Wang et al., 2021). Conversely, Piezo1 knockdown decreases the migration, proliferation and metastasis of human gastric cancer cells by reducing mitochondrial membrane potential and the expression of p53, p21, several cyclin dependent kinases, and VEGF (Wang et al., 2021).

Immunohistochemistry analysis of malignant and benign prostate cancer tissues shows that Piezo1 is elevated with disease progression (Han et al., 2019). Overexpression of Piezo1 promotes tumorigenesis by enhancing Akt/mTOR pathway activation. In particular, Piezo1 promotes cell cycle progression *via* cyclin D1 and cyclin dependent kinase 4 (Han et al., 2019). Accordingly, Piezo1 downregulation in human prostate cancer (DU145) cells reduces proliferation and migration *in vitro* and tumor growth *in vivo* (Han et al., 2019).

RNA sequencing and microarray analyses reveal that Piezo1 expression is correlated with higher grades of glioma and worse clinical outcome (Zhou et al., 2020). Gene ontology analysis shows that high levels of Piezo1 are correlated with tumor microenvironment-related genes that encode proteins involved in ECM organization, angiogenesis and cell migration, including MMPs, mitogen-activated protein kinase (MAPK) family members and PI3K family members (Zhou et al., 2020). Piezo2 is also a crucial regulator of glioma cell growth, migration and invasion. Piezo2 promotes a favorable tumor microenvironment with increased vascular density and leakage by inducing Ca^2+^-dependent Wnt/β-catenin signaling in endothelial cells (Yang et al., 2016). Interestingly, Piezo2 knockdown in murine glioma (GL261) cells is sufficient to abrogate these environmental changes, suggesting a potential cross-talk between glioma and endothelial cells that supports tumor growth and reduces apoptosis (Yang et al., 2016).

Piezo1 also plays a key role in the pathogenesis of OSCC. Immunohistochemistry analysis of surgical specimens shows that Piezo1 expression in OSCC tissues is elevated compared to normal tissues (Hasegawa et al., 2021). Increased YAP activity in human OSCC enhances *Piezo1* mRNA transcript levels (Hasegawa et al., 2021). Piezo1 overexpression subsequently activates ERK1/2 and p38 MAPK pathways through increased Ca^2+^ influx, leading to the enhanced migration and proliferation of OSCC cells (Hasegawa et al., 2021).

Interestingly, both Piezo1 and Piezo2 are downregulated in non-small cell lung cancer (NSCLC) tumor tissue compared to matched adjacent normal tissue (Huang et al., 2019). Accordingly, higher mRNA expression of *Piezo* channels is correlated with better overall survival, especially for patients with lung adenocarcinoma (Huang et al., 2019). Loss of Piezo1 reduces focal adhesion formation and calpain activity, switching normal human bronchial epithelial (16HBE) cells to an ameboid mode of migration (McHugh et al., 2012). Actin cytoskeletal re-arrangement and increased expression of tensin 4 in Piezo1 knockdown cells also results in faster 2D migration speeds and enhanced 3D invasion (McHugh et al., 2012). Additionally, Piezo1 promotes anchorage independent growth leading to formation of more colonies on soft agar (McHugh et al., 2012).

#### TRPC1

TRPC1 is a crucial MIC involved in the regulation of cancer cell migration and metastasis (Canales Coutiño and Mayor, 2021). TRPC1 mediates mechanosensation in response to plasma membrane tension changes, such as pressure and fluid flow (Table 3); however, it is activated through intracellular signaling pathways (Table 2). TRPC1 plays a key role in cell polarity and directed cell migration *via* Ca^2+^ signaling events in the lamellipodium (Fabian et al., 2008). TRPC1 silencing in transformed renal epithelial cells causes a partial loss in cell polarity which impairs the speed and directionality of migrating cells (Fabian et al., 2008). Reduced expression of TRPC1 in CNE2 nasopharyngeal tumor cells also inhibits cell migration and invasion through transwell inserts with 8 μm pores (He et al., 2012). Additionally, TRPC1 knockdown in breast cancer cells reduces ERK1/2 phosphorylation and cell cycle progression (El Hiani et al., 2009; Faouzi et al., 2016). TRPC1 contributes to hypoxia-induced EMT by regulating EGFR phosphorylation, Akt activation, and the expression of HIF-1ɑ, Snai1 and Twist1 (Azimi et al., 2017). Consequently, TRPC1 overexpression is associated with more aggressive breast cancer subtypes and poorer patient outcomes (Azimi et al., 2017). TRPC1 overexpression is also correlated with colorectal cancer progression and poor prognosis (Sun et al., 2021). TRPC1 increases store-operated Ca^2+^ entry in several human colon carcinoma cells through Orai1 and STIM1, leading to enhanced cell proliferation and invasion (Sobradillo et al., 2014). TRPC1 also increases: 1) PI3K/Akt signaling through direct interaction with calmodulin, 2) the expression of CyclinB1 and CDK1 which promote cell-cycle progression through G2, and 3) the expression of mesenchymal markers, such as N-cadherin, Snai1 and Slug (Sun et al., 2021). As a result, colorectal cancer cells expressing TRPC1 proliferate, migrate, invade and metastasize significantly more than cells with TRPC1 knockdown (Sun et al., 2021). Lastly, TRPC1 is overexpressed in human pancreatic cancer cells, where it mediates TGFβ-induced cell migration by increasing intracellular Ca^2+^ release from the endoplasmic reticulum (Dong et al., 2010), and NSCLC cells, where it contributes to disease progression by upregulating HIF-1ɑ through store-operated Ca^2+^ entry (Jiang et al., 2013; Wang et al., 2018a).

#### TRPC5

TRPC5 is another TRP family member that enhances cancer progression (Table 2). Previous studies demonstrate that TRPC5 channels respond to hypoosmotic stimulation and pressure-induced membrane stretch (Table 3), and this mechanosensitivity depends on actin filaments (Gomis et al., 2008; Shen et al., 2015). TRPC5 promotes the initiation, progression and metastasis of colon cancer by inducing EMT *via* HIF1ɑ and Twist1 (Chen et al., 2017a). Colon cancer cells with TRPC5 overexpression exhibit increased intracellular Ca^2+^, which promotes increased migration, invasion and proliferation (Chen et al., 2017a). TRPC5 also regulates the chemoresistance of colon cancer cells by enhancing Wnt/β-catenin signaling, which increases the expression of P-glycoprotein (Wang et al., 2015), an ABC drug efflux transporter, and glucose transporter 1 (GLUT1) (Wang et al., 2017). Patients with poorly differentiated tumors possess CTCs with high levels of TRPC5 expression (Cai et al., 2021). Consequently, TRPC5 overexpression is associated with poorer patient outcomes (Chen et al., 2017a; Cai et al., 2021). TRPC5 also plays an important role in breast cancer chemoresistance. Breast cancer cells resistant to doxorubicin express high levels of TRPC5, which enhances the expression of P-glycoprotein *via* the Ca^2+^-dependent transcription factor NFATc3 (Ma et al., 2012). Interestingly, breast cancer patients contain circulating exosomes with TRPC5 in their peripheral blood (Ma et al., 2014). Ca^2+^ influx *via* TRPC5 enhances the release of these exosomes, which can enter other breast cancer cells and confer resistance (Ma et al., 2014). Additionally, TRPC5 enhances chemoresistance by increasing autophagy *via* calmodulin-dependent protein kinase kinase *β* (CAMKKβ), AMP-activated kinase ɑ (AMPKɑ) and mTOR (Zhang et al., 2017). Accordingly, breast cancer patients exhibit markedly increased levels of TRPC5 and the autophagy marker LC3 after chemotherapy (Zhang et al., 2017). Remarkably, lentiviral injection of an shRNA against TRPC5 at tumor sites reverses chemoresistance to doxorubicin and paclitaxel resulting in tumor regression (Ma et al., 2012). Thus, TRPC5 may be an important clinical target for the treatment of breast cancer. Epidermal growth factor signaling *via* PI3K, Rac1 and phosphatidylinositol 4-phosphase 5-kinase (PIP(5)Kɑ) enhances TRPC5 trafficking to the plasma membrane in human embryonic kidney (HEK-293) cells (Bezzerides et al., 2004). This pathway may participate in the translocation and release of TRPC5 exosomes in breast cancer, however further investigation is required.

#### TRPM2

TRPM2 also enhances cancer cell migration and metastasis (Table 2). TRPM2 can be activated by temperature (Tan and McNaughton, 2016), oxidative stress (Perraud et al., 2005), and other stimuli that increase NAD^+^-related metabolites (Table 3) (Huang et al., 2018). H_2_O_2_-mediated activation of TRPM2 in cervical cancer (HeLa) and prostate cancer (PC-3) cells induces actin cytoskeleton remodeling, focal adhesion disassembly, and enhanced cell migration (Li et al., 2016). Interestingly, this phenotype is mediated by intracellular release of Zn^2+^ rather than Ca^2+^ (Li et al., 2016). Overexpression of TRPM2 is also associated with poor outcomes in pancreatic cancer patients due to PKC/MEK pathway activation, which results in increased cell proliferation, migration and invasion (Lin et al., 2021). Furthermore, TRPM2 enhances the migration, invasion and tumorigenesis of gastric cancer cells by decreasing PTEN activity and increasing Akt signaling (Almasi et al., 2019). These changes downregulate E-cadherin expression and upregulate EMT-related genes *Twist* and *Zeb1* (Almasi et al., 2019). Additionally, TRPM2 reduces apoptosis and enhances mitochondrial metabolism, which increases cell proliferation, autophagy and mitophagy through a c-Jun N-terminal kinase (JNK)-dependent signaling pathway (Almasi et al., 2018). Due to its effects on autophagy, TRPM2 downregulation sensitizes gastric cancer cells to paclitaxel and doxorubicin treatment (Almasi et al., 2018).

#### TRPM4

TRPM4 is another TRPM channel subfamily member associated with cell migration and cancer metastasis. Although TRPM4 itself is not a stretch-activated Ca^2+^ channel (Table 2), it may work in tandem with other MICs to facilitate mechanosensing (Table 3). For example, TRPM4 acts together with Piezo1 to sense pressure overload in left ventricular hypertrophy (Yu et al., 2022). In mouse embryonic fibroblasts, TRPM4 localizes to adhesions and regulates cell spreading, migration and invasion by altering focal adhesion and actin cytoskeleton dynamics (Cáceres et al., 2015). Interestingly, TRPM4 mediates FAK and Rac GTPase activation following serum-induced increases in intracellular Ca^2+^ (Cáceres et al., 2015). One possible explanation is that TRPM4 facilitates localized Na^+^ influx, which causes membrane depolarization and leads to the activation of voltage-dependent Ca^2+^ channels (Cáceres et al., 2015). TRPM4 also regulates the migration and invasion of prostate cancer (PC-3) cells by altering the expression of genes involved in EMT, including the repression of E-cadherin and upregulation of Snai1, MMP-9, N-cadherin and vimentin (Sagredo et al., 2019). Accordingly, TRPM4 overexpression is correlated with higher grade prostate cancer (Sagredo et al., 2019). Similarly, knockout of TRPM4, which is overexpressed in human colorectal tumors and correlates with late-stage metastatic cancer, hinders cell migration, invasion, proliferation and viability (Kappel et al., 2019). In contrast, increased expression of TRPM4 in endometrial carcinoma cells is associated with a more favorable prognosis (Li et al., 2020b). TRPM4 downregulation due to estrogen decreases p53 levels, promotes PI3K/Akt/mTOR pathway activation, and increases EMT progression (Li et al., 2020b). Consequently, TRPM4 knockdown increases the migratory ability of endometrial carcinoma (AN3 CA) cells.

#### TRPM7

TRPM7 is a divalent cation channel with inherent serine/threonine kinase activity (Table 2) that is ubiquitously expressed in the human body and involved in many (patho) physiological processes. Previous studies indicate that TRPM7 is activated by hydraulic pressure (Zhao et al., 2019; Zhao et al., 2021), shear stress (Yankaskas et al., 2021) and cell swelling (Numata et al., 2007) (Table 3). TRPM7 can mediate breast cancer cell migration and invasion through Src and MAPK signaling pathways, without the involvement of Akt (Meng et al., 2013). In addition, TRPM7 regulates cell-cell adhesions, cell-matrix adhesions and cell migration speeds by regulating myosin II-based contractility (Middelbeek et al., 2012). For these reasons, TRPM7 expression is functionally required to form metastases in mouse xenograft models and predicts poor outcome in breast cancer patients (Middelbeek et al., 2012). In ovarian cancer, TRPM7 promotes the phosphorylation of Akt, Src and p38 (Wang et al., 2014) and induces EMT *via* Twist1 expression (Liu et al., 2019b). Conversely, TRPM7 depletion inhibits cell proliferation, colony formation, migration, invasion and metastasis (Wang et al., 2014; Liu et al., 2019b). As a result, TRPM7 overexpression is associated with poor prognosis in ovarian cancer patients as well (Wang et al., 2014). In prostate cancer, cholesterol stimulates Ca^2+^ influx through TRPM7, which enhances cell proliferation and migration *via* ERK and Akt phosphorylation, reduces E-cadherin expression, and increases calpain activity (Sun et al., 2014a). Accordingly, TRPM7 knockdown reverses EMT, leading to the downregulation of MMPs and upregulation of E-cadherin (Chen et al., 2017b). Androgen-independent prostate cancer cells also undergo EMT in response to hypoxia *via* a TRPM7/HIF-1ɑ/Annexin A1 signaling pathway (Yang et al., 2020a). In contrast, TRPM7 knockdown suppresses cell migration, invasion and EMT by enhancing RACK1-mediated degradation of HIF-1α (Yang et al., 2020a). Consequently, TRPM7 overexpression is associated with poor survival in prostate cancer patients (Yang et al., 2020a). TRPM7 expression is also associated with advanced colorectal cancer due to EMT (Su et al., 2019). TRPM7 downregulation reverses EMT, which promotes apoptosis and reduces cell proliferation, migration, and invasion (Su et al., 2019). On the other hand, TRPM7 enhances the invasion of pancreatic adenocarcinoma cells through an HSP90ɑ/uPA/MMP-2 proteolytic axis (Rybarczyk et al., 2017). This axis may enhance cell migration in response to elastin-derived peptides that are released following ECM degradation (Lefebvre et al., 2020). As a result, TRPM7 is inversely correlated with the survival of pancreatic cancer patients (Rybarczyk et al., 2017). TRPM7 is also overexpressed in bladder cancer and promotes cell migration, invasion and proliferation which results in poor patient outcomes (Gao et al., 2017). Finally, sustained Ca^2+^ influx through TRPM7 enhances the migratory ability of human nasopharyngeal carcinoma cells by stimulating ryanodine receptors to release additional Ca^2+^ from intracellular stores (Chen et al., 2010).

#### TRPV2

TRPV2 was initially characterized as a heat-gated ion channel (Caterina et al., 1999; Liu and Qin, 2016); however, it can also respond to membrane stretch in neurons (Table 2) (Mihara et al., 2010; Shibasaki et al., 2010). In addition, TRPV2 function and expression are linked to cell migration and cancer metastasis (Table 3). TRPV2 enhances the migration, invasion proliferation and survival of esophageal squamous cell carcinoma cells (Kudou et al., 2019). Accordingly, TRPV2 expression is associated with poor patient prognosis (Kudou et al., 2019). In addition, TRPV2 increases gastric cancer cell migration and invasion through the TGFβ signaling pathway (Kato et al., 2022). In contrast, TRPV2 knockdown reduces the expression of MMP-2, MMP-9 and integrin alpha V (Kato et al., 2022). As a result, TRPV2 expression is associated with lymphatic invasion, venous invasion and poor prognosis in gastric cancer patients (Kato et al., 2022). Interestingly, several studies indicate that TRPV2 facilitates the uptake of chemotherapy drugs. Moreover, cannabidiol can be used to trigger TRPV2 to enhance the uptake of chemotherapy drugs *via* increased Ca^2+^ influx. In particular, cannabidiol has been shown to sensitize breast cancer (Elbaz et al., 2018), endometrial cancer (Marinelli et al., 2020) and glioblastoma (Nabissi et al., 2013) cells to chemotherapeutic agents.

#### TRPV4

TRPV4 is another TRPV channel subfamily member with well-characterized roles in cell migration and cancer metastasis. Like many other TRP channels, TRPV4 is a polymodal protein (Table 2) that can be activated by cell swelling, shear stress, moderate temperatures (∼27°C), hypoosmotic conditions and chemical agonists (Clapham, 2003; Liedtke et al., 2003; Mendoza et al., 2010) (Table 3). TRPV4 regulates the migration of several cell types by modulating focal adhesion dynamics (Mrkonjić et al., 2015). Ca^2+^ influx through TRPV4 at focal adhesion sites activates calpain, which promotes focal adhesion disassembly and efficient retraction of the trailing edge (Mrkonjić et al., 2015). In contrast, overexpressing TRPV4 mutants that lack the phosphoinositide-binding site (^121^AAWAA) or a functional pore (TRPV4-M680D) reduces calpain activity, increases focal adhesion size, and promotes the formation of a tail, which anchors the cell to the substrate (Mrkonjić et al., 2015). TRPV4 overexpression is correlated with significantly poorer overall survival and disease-free survival in breast cancer patients (Lee et al., 2017a). Ca^2+^ influx through TRPV4 enhances Akt and FAK phosphorylation, reduces E-cadherin and *β*-catenin expression, and alters the expression of many other proteins involved cytoskeleton and ECM remodeling (Lee et al., 2017a). Indeed, TRPV4 overexpression promotes actin reorganization, cell blebbing and cell softness (Lee et al., 2016). These changes increase transendothelial migration and lung metastasis of breast cancer cells (Lee et al., 2016; Lee et al., 2017a). TRPV4 also accelerates glioma cell migration and invasion through Akt phosphorylation and Rac1 activation (Ou-Yang et al., 2018). Moreover, TRPV4 can promote the formation of filopodia through Cdc42 and N-wasp (Yang et al., 2020b). TRPV4 inhibition reduces tumor growth, decreases invasion into the surrounding brain tissue, and significantly prolongs the survival time of mice (Yang et al., 2020b). Accordingly, TRPV4 overexpression is associated with poor prognosis in glioma patients (Ou-Yang et al., 2018; Yang et al., 2020b). In line with these studies, TRPV4 overexpression increases the migration and invasion of colon cancer cells by activating Akt (Zhang et al., 2021). Silencing or inhibiting TRPV4 suppresses invasiveness by abrogating ZEB1-mediated EMT (Zhang et al., 2021). Finally, TRPV4-mediated Ca^2+^ influx in endometrial cancer cells enhances cell migration *via* a RhoA/ROCK1/LIMK/cofilin pathway that remodels the actin cytoskeleton and reinforces focal adhesions (Li et al., 2020c).

## Concluding remarks and future outlook

In the last two decades, physical cues have been identified as crucial regulators of cancer progression (Wirtz et al., 2011; Nia et al., 2020). More recently, the role of extracellular fluids in regulating cell behavior has also emerged (Follain et al., 2020). Cells encounter both solid and fluid interfaces *in vivo* (Figure 1) and thus it is crucial to understand how cells interpret physical cues to better control metastatic spread. MICs play an important role in sensing and transducing physical stimuli, such as compression, membrane tension, heat, pressure, hydraulic resistance, shear stress, substrate stiffness and viscoelasticity (Table 1), which allows cells to adapt to the local microenvironment. While the role of MICs in normal cells is well known (Ranade et al., 2015), their expression and function in cancer cells has recently received attention (Adapala et al., 2016; Dombroski et al., 2021; Karki and Tojkander, 2021; Yankaskas et al., 2021). Of particular interest is how MICs allow cancer cells to sense, integrate and interpret physical cues encountered during metastasis. Here, we described physical cues encountered at the primary tumor site followed by those encountered during invasion and inside the vasculature. We provided an overview of Piezo1/2, TRPC1, TRPC5, TRPM2, TRPM4, TRPM7, TRPV2 and TRPV4 (Table 2) and showed that these channels participate in numerous biochemical pathways that enhance metastatic phenotypes, such as cell migration, invasion and metastasis (Table 3).

The classical view of mechanosensing implicates focal adhesions (Box 1) as the primary sensors involved in perceiving and transmitting physical cues from the substrate to the cellular cytoskeleton (Albiges-Rizo et al., 2009; Geiger et al., 2009; Plotnikov et al., 2012; Horton et al., 2016; Stutchbury et al., 2017). However, accumulating evidence demonstrates that the dynamics of these critical structures are strongly influenced by the activity of MICs. For example, Piezo1 can localize to focal adhesions and regulate their assembly *via* FAK (Chen et al., 2018). Piezo1 can also activate Rho GTPase family members, alter the expression of integrin subunits, and induce EMT (Zhang et al., 2018; Wang et al., 2021). Similarly, Piezo2 can regulate focal adhesion dynamics *via* Fyn kinase and calpain activity and promote stress fiber assembly through RhoA/mDia (Pardo-Pastor et al., 2018). TRP channels have also been shown to regulate focal adhesion dynamics by activating calpain, FAK and PI3K/Akt signaling (Sun et al., 2014a; Wang et al., 2014; Cáceres et al., 2015; Mrkonjić et al., 2015; Lee et al., 2017a; Azimi et al., 2017; Ou-Yang et al., 2018; Almasi et al., 2019; Li et al., 2020b; Sun et al., 2021; Zhang et al., 2021). For instance, TRPV4 knockdown reduces calpain activity, which prevents focal adhesion disassembly and leads to the formation of large adhesions that anchor the cell (Mrkonjić et al., 2015). Moreover, TRP channels can regulate actin dynamics and stimulate cellular contractility by activating Rac, RhoA/myosin-II, RhoA/ROCK1/LIMK/cofilin or calmodulin/IQGAP/Cdc42 (Adapala et al., 2016; Li et al., 2020c; Yankaskas et al., 2021). Additionally, TRPC1, TRPC5, TRPM2, TRPM4, TRPM7, TRPV2 and TRPV4 can independently induce EMT in cancer cells (Chen et al., 2017a; Azimi et al., 2017; Chen et al., 2017b; Almasi et al., 2019; Liu et al., 2019b; Kudou et al., 2019; Sagredo et al., 2019; Su et al., 2019; Yang et al., 2020a; Li et al., 2020b; Zhang et al., 2021; Kato et al., 2022).

Cells on 2D substrates can freely spread out and migrate without any physical perturbations to their plasma membrane. On the other hand, cells migrating through 3D ECM frequently encounter obstacles that deform the plasma membrane, causing local changes in tension. Additionally, as we described in this article, tumor cells continually experience forces arising from bodily fluids at different locations in the metastatic cascade which cannot be directly detected by focal adhesion complexes. Thus MICs, together with focal adhesion components, can facilitate effective cell migration/invasion in response to changing mechanical cues. At the same time, traction forces generated by actomyosin contractility may also cause local changes in membrane tension surrounding adhesions (Ellefsen et al., 2019). These changes may induce MIC channel opening, which subsequently regulates adhesion strength and actomyosin contractility. Therefore, there is likely a dynamic crosstalk between adhesions and MICs that requires further investigation.

MICs may also regulate the assembly of specialized invasive structures known as invadopodia (Box 2), which contain a perpendicular alignment of actin filaments with respect to the underlying ECM. These structures allow cells to exert protrusive forces on the matrix as MMPs enzymatically degrade fibers (Albiges-Rizo et al., 2009). Focal adhesions and invadopodia share similar scaffolding and signaling components, albeit in a different arrangement (Revach et al., 2020). Given that Ca^2+^ influx through MICs regulates focal adhesion formation, it is plausible that MICs may regulate invadopodia formation. Indeed, Piezo1 and Piezo2 have recently been shown to enhance invadopodia formation in breast cancer cells (Pardo-Pastor et al., 2018; Luo et al., 2022). Alternatively, MIC crosstalk with intracellular store-operated Ca^2+^ (SOC) channels may control invadopodia formation. In melanoma cells, the SOC channel Orai1 localizes to invadopodia and spontaneously mediates discrete Ca^2+^ transients (Lu et al., 2019). Ca^2+^ entry through Orai1/STIM1 initiates calmodulin binding to the autoinhibitory domain of Pyk2, which allows Pyk2 to activate Src (Lu et al., 2019). Overexpression of a dominant-negative Orai1 mutant (Orai1-E106A) or STIM1 knockout largely abolishes invadopodial Ca^2+^ signals (Lu et al., 2019). Store-operated Ca^2+^ entry blockade also inhibits invadopodia formation and ECM degradation (Sun et al., 2014b). Accordingly, STIM1 knockdown significantly reduces the metastatic ability of melanoma cells (Sun et al., 2014b). In contrast, TRPM7 knockout fails to affect the frequency, amplitude or duration of Ca^2+^ signals in invadopodia (Lu et al., 2019). By responding to substrate stiffness, MICs, such as Piezo1/2 and TRPV4, may also regulate invadopodia formation in response to matrix rigidity. At low matrix stiffness (0.165 kPa), cells use membrane blebs to invade in a protease-independent manner (Aung et al., 2014). In contrast, higher matrix stiffness induces the formation of invadopodia to facilitate protease-dependent invasion (Aung et al., 2014). Several studies demonstrate that invadopodia form optimally in response to certain substrate stiffnesses. For example, breast cancer cells form more invadopodia as substrate stiffness increases from 1.1 to 28 kPa but exhibit less invadopodia on 3.1 and 5.6 MPa substrates (Aung et al., 2014; Dalaka et al., 2020). Similarly, head and neck squamous cell carcinoma cells degrade ECM more effectively on 22.7 kPa compared to 1.0 kPa (Jerrell and Parekh, 2014). Thus, it would be interesting to determine whether MIC function is required for invadopodia formation in response to these stiffnesses.

Box 2 **| Invadopodia are important structures that facilitate cancer cell invasion. Invadopodia are dynamic structures with a typical lifetime of 5–15 min on 2D substrates (Eddy et al., 2017). Current models of formation suggest that cofilin, Arp2/3, N-WASP and cortactin are involved in precursor core initiation (Eddy et al., 2017). Tks5 and SHIP2 are subsequently recruited to stabilize the precursor core to the plasma membrane (Eddy et al., 2017). Finally, Nck1 and Cdc42 activate the N-WASP-Arp2/3 complex to nucleate actin polymerization; Cdc42 and RhoA also recruit membrane tethered matrix metalloproteinase (MT1-MMP) to the plasma membrane (Eddy et al., 2017). Invadopodia allow cancer cells to breach basement membranes, intravasate into and extravasate out of the bloodstream, and establish metastatic colonies (Leong et al., 2014; Harney et al., 2015; Eddy et al., 2017). Accordingly, knockdown of critical invadopodia components, such as Tks5, cortactin, and LPP, reduces cancer cell proliferation, invasion and metastasis (Blouw et al., 2015; Ngan et al., 2017).**

Changes in membrane tension are thought to activate MICs and trigger Ca^2+^ current across the cell surface (Matthews et al., 2006). Indeed, each of the mechanical forces described above enhance intracellular Ca^2+^ levels, which stimulate intracellular signaling pathways involved in cell migration and invasion. In addition to direct mechanical activation, the activity of MICs may be influenced by ion channels and transporters that affect plasma membrane tension (Box 3). For example, breast cancer cells can migrate through tightly confined channels by preferentially polarizing ion transporters and water channels in a process known as the Osmotic Engine Model (OEM) (Stroka et al., 2014a; Stroka et al., 2014b; Li and Sun, 2018; Li et al., 2019b). Na^+^/H^+^ exchanger isoform-1 (NHE1) and aquaporin 5 (AQP5) polarization to the leading edge of cells facilitates water influx at the cell front, which promotes forward movement of the leading-edge membrane (Stroka et al., 2014a). In contrast, hypotonic shock at the cell front reverses the direction of cell migration by forcing cells to expel water at their “old” leading edge (Stroka et al., 2014a). Such expansion-contraction events can accentuate or attenuate membrane tension in migrating cells, which may trigger MIC activation and lead to a feedback loop between volume regulatory ion channels and MICs. TRP Ca^2+^ currents are also affected by changes in the transmembrane voltage (Zheng, 2013), which may be altered due to ion fluxes through other ion channels/transporters. Thus, it is expected that MICs should have a broad role in cell mechanosensation as well as sensing of cell electrical activity.

Box 3 **| Ion channels that affect membrane tension may influence MIC activation. Cells possess feedback mechanisms that actively maintain membrane tension at a homeostatic level. In the unperturbed state, cell membrane tension arises from balancing osmotic and hydraulic pressure differences across the cell surface (Li et al., 2020a). From the Young-Laplace relation, the hydraulic pressure difference across the cell surface at mechanical equilibrium, ΔP, is balanced by tension in the cell surface, i.e., ΔP=T/R , where T is the cell surface tension and R is the mean surface curvature. The tension in the cell surface, however, is a combination of membrane tension, T_m_, and tension in the actomyosin cortex, T_a_: $T=T_{m}+T_{a}$ , and $T_{a}=\sigma h$ , where h is the thickness of the cell cortex and σ is the cortical stress. Since the cortex is much thicker than the membrane, cortical tension is much larger than membrane tension ( $T_{a}\gg T_{m}$ ). Moreover, cortical tension can be balanced by actomyosin contraction, and is therefore actively controlled by the cell. Thus, when membrane tension increases, Ca^2+^ flux through opened MICs triggers actomyosin remodeling and an increase in myosin activity, such that membrane tension is reduced over the time scale of minutes (Tao and Sun, 2015). Over longer time scales, membrane tension changes will also alter cell endocytic activity and membrane trafficking from membrane reservoirs such as the endoplasmic reticulum (Sens and Turner, 2006). Therefore, membrane tension in mammalian cells is likely actively maintained and controlled by the actomyosin system and membrane trafficking. As such, membrane tension changes and TRP currents are likely transient, with spikes occurring during cell mechanosensation.**

Taken together, MICs bestow cancer cells with the capacity to sense and respond to complex physical cues encountered during metastasis. As tumor cells migrate through the tissue microenvironments, they rely on mechanotransduction pathways engaged by MICs to choose the most efficient migratory route and adjust cellular functions accordingly. Interestingly, the expression level of MICs may be stage dependent, as demonstrated by TRPM7. On one hand, TRPM7 overexpression enhances cancer cell migration *via* myosin II-based contractility (Middelbeek et al., 2012; Meng et al., 2013), however, TRPM7 downregulation is required during intravasation to de-sensitize cells to shear stress (Yankaskas et al., 2021). Stage-dependent expression may allow cancer cells to dynamically refine their mechanical properties, which is required to successfully complete all steps of the metastatic cascade (Gensbittel et al., 2021). Thus, a detailed understanding and characterization of physical forces and associated MICs will provide opportunities to improve the treatment of metastatic cancer.
